# Siweixizangmaoru Decoction Alleviates Rheumatoid Arthritis by Enhancing Pol β-Mediated Attenuation of DNA Damage and Suppression of cGAS/STING/NF-κB/NLRP3-Associated Pyroptosis

**DOI:** 10.3390/ph19081302

**Published:** 2026-08-18

**Authors:** Xiaotong Chu, Xiao Chen, Yanxiang Yuan, Yanfei Niu, Wentao Zhou, Yiming Cui, Yongyue Pan, Haifeng Liu, Zhuoma Dongzhi, Shan Huang, Bin Li

**Affiliations:** 1 Department of Pharmaceutical Engineering and Pharmaceutical Chemistry, School of Chemical Engineering, Qingdao University of Science and Technology, Qingdao 266042, China; 17205228560@163.com (X.C.);; 2 Department of Pharmacy, College of Medicine, Xizang University, Lhasa 850000, Chinazmdz1980@utibet.edu.cn (Z.D.); 3 College of Nursing, Qingdao Binhai University, Qingdao 266555, China

**Keywords:** rheumatoid arthritis, Siweixizangmaoru decoction, DNA polymerase β, pyroptosis, network pharmacology

## Abstract

**Objective:** Siweixizangmaoru decoction (SXD), a classical Tibetan prescription documented in the medical text *Four Medical Tantras*, has shown therapeutic activity in experimental rheumatoid arthritis (RA). However, its candidate active constituents and underlying mechanisms remain unclear. **Methods:** In this study, serum-absorbed constituents following SXD administration were profiled using ultra-high-performance liquid chromatography coupled with quadrupole-Orbitrap high-resolution mass spectrometry (UHPLC-Q-Orbitrap HRMS), and network pharmacology was employed to identify core targets and candidate active constituents of SXD against RA. Candidate active constituents were further screened using cell-based assays, and the selected constituents were quantified using HPLC fingerprint analysis. Both in vivo (CIA rat model) and in vitro (RAW264.7 pyroptosis model) systems were used to investigate the pharmacological effects and mechanisms of SXD. **Results:** A total of 11 absorbed constituents were identified in the serum of SXD-treated rats, and four preliminary candidate active constituents, including chebulagic acid, kaempferol, gentiopicroside, and berberine, were screened. The combined in vivo and in vitro results showed that SXD attenuated RA progression and reduced inflammatory cytokine levels in rat serum and cell culture supernatants. At the molecular level, SXD increased Pol β expression and reduced DNA damage markers, cytosolic dsDNA accumulation, and cGAS/STING-, NF-κB-, and NLRP3-associated signaling. Pol β knockdown partially reduced the effects of SXD. **Conclusions:** These findings support the partial involvement of Pol β-associated processes in the anti-arthritic effects of SXD.

## 1. Introduction

Rheumatoid arthritis (RA) is a chronic autoimmune disorder in which chronic synovial inflammation gradually drives cartilage and bone destruction and ultimately impairs joint function [1]. Beyond the joints, RA is frequently accompanied by systemic inflammatory and multiple comorbidities, which together reduce patients’ quality of life and long-term prognosis [2]. Ongoing synovial inflammation drives immune-cell infiltration, synovial proliferation, and excessive production of pro-inflammatory cytokines, making it a central event in RA progression [3]. Pyroptosis, an inflammatory type of programmed cell death, has recently become an important focus in RA research [4]. This process typically involves gasdermin D (GSDMD)-dependent membrane pore formation together with the release of IL-18 and IL-1β [5]. Studies have shown that GSDMD is highly activated in the synovial tissues of patients with RA, and the activation level of the NLRP3/Caspase-1 pathway is positively correlated with DAS28 scores and the bone destruction index [6].

Increasing evidence suggests that persistent DNA damage and impaired DNA repair capacity may aggravate RA by activating cGAS/STING, NF-κB, and NLRP3 pathways, thereby promoting pyroptosis and inflammation [7]. Consistently, increased γH2AX, a marker of DNA double-strand breaks (DSBs), and decreased DNA polymerase β (Pol β) have been observed in patients with RA, suggesting that genomic instability drives disease progression through coordinated activation of inflammatory and pyroptotic pathways [8,9]. Pol β is a key BER enzyme that maintains genomic stability by repairing base lesions and single-strand breaks. These findings highlight DNA damage and repair dysfunction as potential therapeutic targets for RA. However, the use of NSAIDs, DMARDs, and glucocorticoids is often restricted by incomplete efficacy and adverse reactions such as gastrointestinal discomfort and organ toxicity [10,11]. Accordingly, considerable research interest has been devoted to exploring potential therapeutic options from traditional medicine.

The special high-altitude environment of the Qinghai–Tibet Plateau is associated with a relatively high RA burden in that region [12], which has contributed to the extensive clinical experience of traditional Tibetan medicine (TTM) in RA. Within the theoretical system of TTM, RA is considered a type of “Huangshui disease”, and its pathogenesis is attributed to the imbalance of “Huangshui” caused by environmental and dietary factors. In recent years, classical Tibetan medicinal prescriptions for the treatment of RA have attracted increasing attention. Studies suggest that Wuwei Leze powder can mitigate synovial inflammation and cartilage damage by suppressing pro-inflammatory cytokine production and inhibiting NF-κB pathway activation [13]; Qi-Sai-Er-Sang-Dang-Song Decoction exerts anti-inflammatory effects mainly by regulating T cells, suppressing macrophage-mediated inflammatory responses, and inhibiting NF-κB/MAPK signaling [14]; and Wuweiganlu mitigates synovial hyperplasia and bone erosion by modulating oxidative stress, downregulating inflammatory mediators, and inhibiting osteoclast differentiation [15].

Siweixizangmaoru decoction (SXD), a traditional Tibetan medicinal formula used for the treatment of RA, is documented in the Tibetan medical classic *Four Medical Tantras*, where it is described as having the functions of “cooling the blood” and “astringing dry Huangshui” [16]. SXD comprises *Berberis thunbergii* DC. (BT, Xiaobo), *Gentiana macrophylla* Pall. (GM, Qinjiao), *Rhamnella gilgitica* Mansf. et Melch (RG, Xizangmaoru), and *Terminalia chebula* Retz. (TC, Hezi). Modern pharmacological studies of its constituent herbs have focused mainly on anti-inflammatory, immunomodulatory, and antioxidant effects, with GM [17] and TC [18,19] among the most extensively investigated in arthritis-related models. Previous studies from our group showed that SXD alleviated experimental arthritis [20,21]. However, its candidate active constituents and underlying molecular mechanisms remain incompletely defined.

Whereas our previous studies focused on JAK2/STAT3 and NF-κB signaling or on the gut microbiota, SCFA restoration, and immunomodulation, the present study addresses a distinct mechanistic question. We investigated whether the effects of SXD in RA are associated with changes in Pol β expression, DNA damage, cytosolic dsDNA accumulation, signaling through cGAS/STING, NF-κB, and NLRP3, and pyroptosis. Chemical profiling of constituents detected in serum, network pharmacology, cellular and CIA rat experiments, and Pol β knockdown were integrated to examine this relationship. Candidate active constituents were also preliminarily screened, providing a chemical basis for future quality evaluation and development of SXD.

## 2. Results

### 2.1. Analysis of Absorbed Constituents in Rat Serum

**Figure 1:**
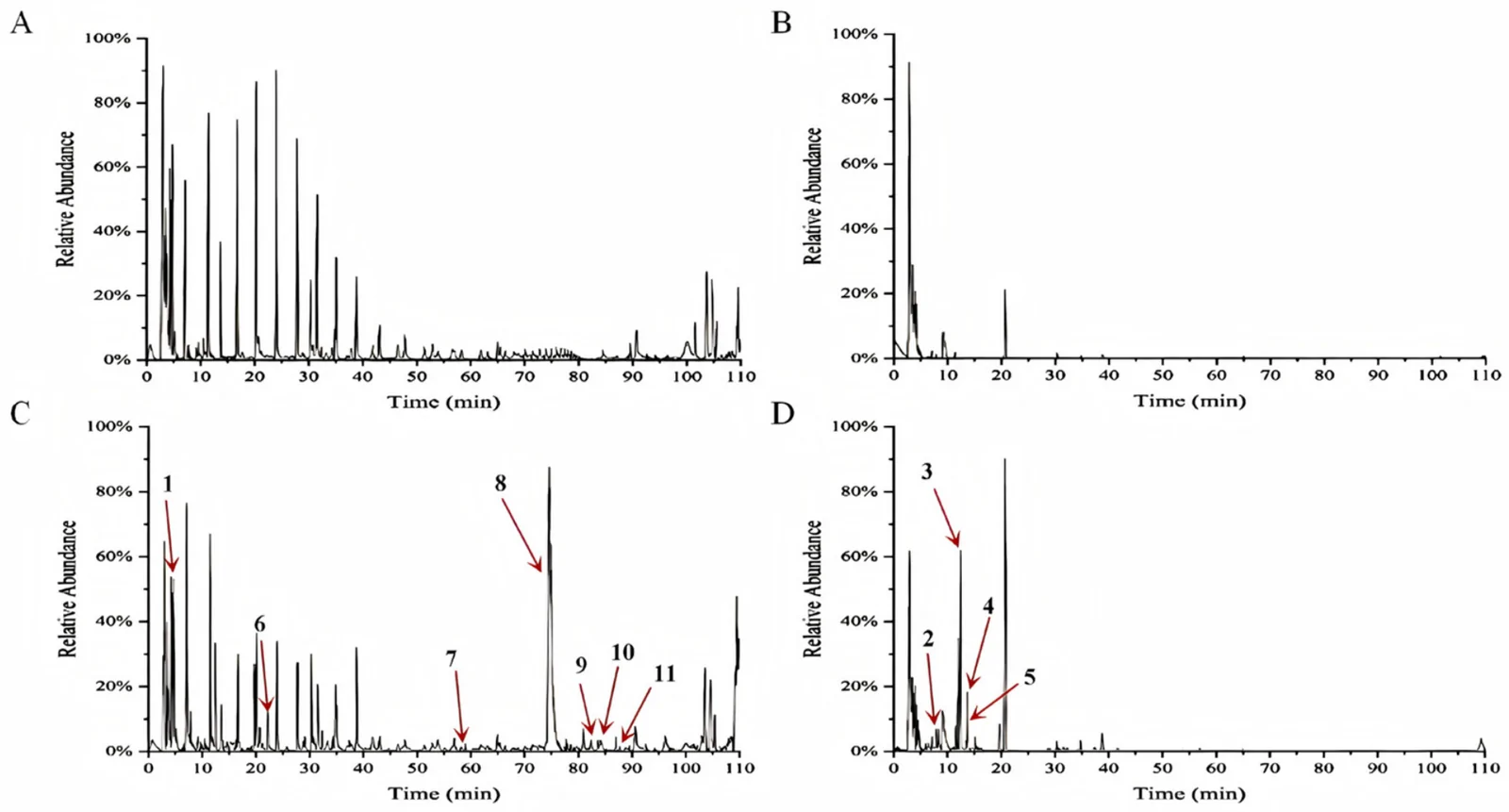
Total ion chromatograms of blank and medicated serum samples. (**A**,**B**) Blank serum in positive- and negative-ion modes, respectively; (**C**,**D**) medicated serum in positive- and negative-ion modes, respectively.

Total ion chromatograms of blank and medicated serum in positive- and negative-ion modes are shown in Figure 1. Comparison with previously characterized SXD constituents, together with accurate-mass and fragmentation data, supported the tentative identification of 11 prototype compounds in rat serum (Table 1). Their chemical structures are shown in Figure S1. The absorbed constituents of SXD mainly included alkaloids, phenolic acids, iridoid glycosides, and flavonoids, which are known to exert anti-inflammatory, immunomodulatory, and anti-arthritic activities.

### 2.2. Comprehensive Network Pharmacology Analysis of SXD in Relation to RA

**Figure 2:**
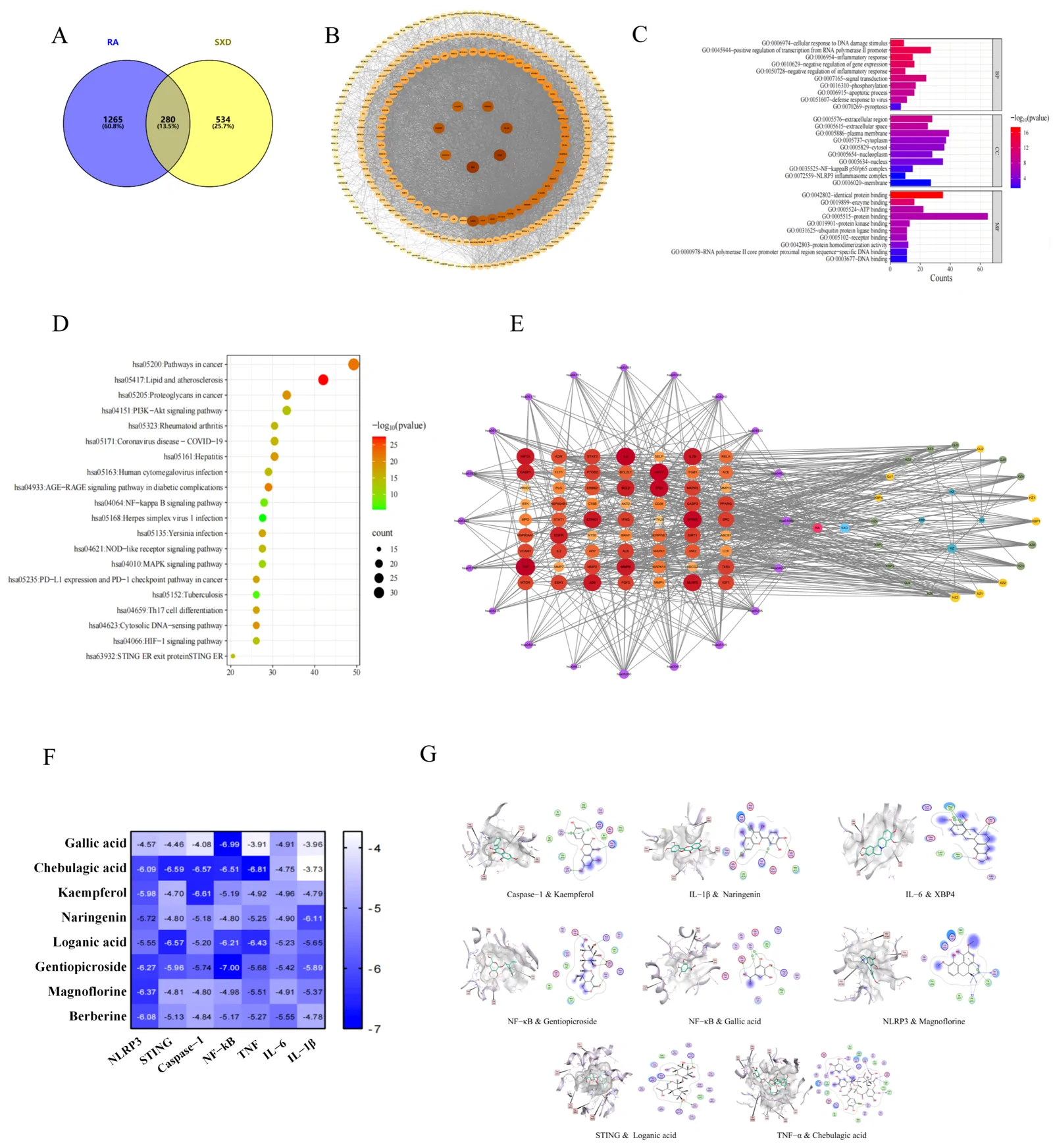
Comprehensive network pharmacology analysis of SXD in relation to RA. (**A**) Venn diagram of targets associated with SXD components and RA. (**B**) Protein–protein interaction (PPI) network and core targets for SXD against RA. (**C**) Bar graph of Gene Ontology (GO) enrichment analysis. (**D**) Bubble chart of Kyoto Encyclopedia of Genes and Genomes (KEGG) enrichment analysis. (**E**) Interactive “Herbs-Components-Targets-Pathways” network. Red circles represent core targets and RA; blue icons represent SXD and its constituent herbs; orange-yellow circles represent the top 8 constituents by degree value; dark green represents other constituents. (**F**) Heatmap analysis of binding energy. (**G**) Visualization of molecular docking between SXD’s active constituents and core targets (showing the targets with the highest binding energy for the 8 candidate active constituents of SXD).

Based on the absorbed constituents identified in rat serum and our previous studies, 534 potential targets were predicted using public databases. Meanwhile, 1265 RA-related targets were retrieved from disease databases, and 280 overlapping targets were obtained by intersection analysis (Figure 2A). These targets were used to build a PPI network, followed by GO and KEGG enrichment analyses. With a threshold of *p* < 0.05, 140 KEGG pathways were identified, among which the top 20 pathways ranked by count value were selected. The enrichment results pointed mainly to NF-κB, PI3K-Akt, AGE-RAGE, cGAS/STING, NLRP3, and MAPK signaling pathways (Figure 2B–D).

Furthermore, a “drug-component-disease-target” network was constructed (Figure 2E), and the top eight compounds with the highest degree values were considered the candidate active constituents of SXD, including gallic acid, naringenin, chebulagic acid, gentiopicroside, kaempferol, loganic acid, magnoflorine, and berberine. Notably, these eight components were also detected among the prototype compounds absorbed into the serum of SD rats. As shown in Figure 2E, these eight candidate active constituents exhibited close associations with key targets, including NLRP3, NF-κB, IL-1β, Caspase-1, and STING (indicated by larger node size and darker color).

Molecular docking analysis was conducted to further assess the binding interactions between eight major compounds and seven core targets (Figure 2F). All eight compounds showed docking scores below −4 kcal/mol, suggesting favorable predicted interactions with the targets (Figure 2G).

### 2.3. Screening and Quantification of Selected Candidate Active Constituents in SXD

To preliminarily identify candidate constituents that may contribute to the anti-inflammatory activity of SXD, the effects of eight candidate components on RAW264.7 cells were evaluated (Figure S2), and subsequent experiments were performed at 20 μM. The eight components significantly reduced NO and inflammatory cytokine levels (IL-6, TNF-α, IL-1β, and IL-18) compared with the model group. Chebulagic acid, kaempferol, gentiopicroside, and berberine produced the most consistent anti-inflammatory responses and were selected for follow-up study.

In the established chromatographic fingerprint, 20 common peaks were identified (Figure S3), among which 13 peaks were further characterized by comparison with reference standards (Figure S4). Among them, peak 8 was identified as gentiopicroside, peak 13 as chebulagic acid, peak 15 as berberine hydrochloride, and peak 19 as kaempferol. The precision, repeatability, accuracy, and chromatographic similarity of the HPLC fingerprinting method were evaluated using the four selected constituents. The RSD values of peak areas were all <1.04%, and those of component contents were <1.98%. The average recoveries ranged from 99.03% to 99.74% with RSD values < 1.22%, indicating good accuracy (Table S4). The similarities between chromatograms of 20 batches of SXD and the reference were all >0.95 (Table S5), indicating low variation and good stability. This suggests that the fingerprint is a reliable tool for quality evaluation of SXD. The contents of the preliminary candidates are summarized in Table S6.

### 2.4. SXD and MIX Attenuate LPS/ATP-Induced Pyroptosis in RAW264.7 Cells

**Figure 3:**
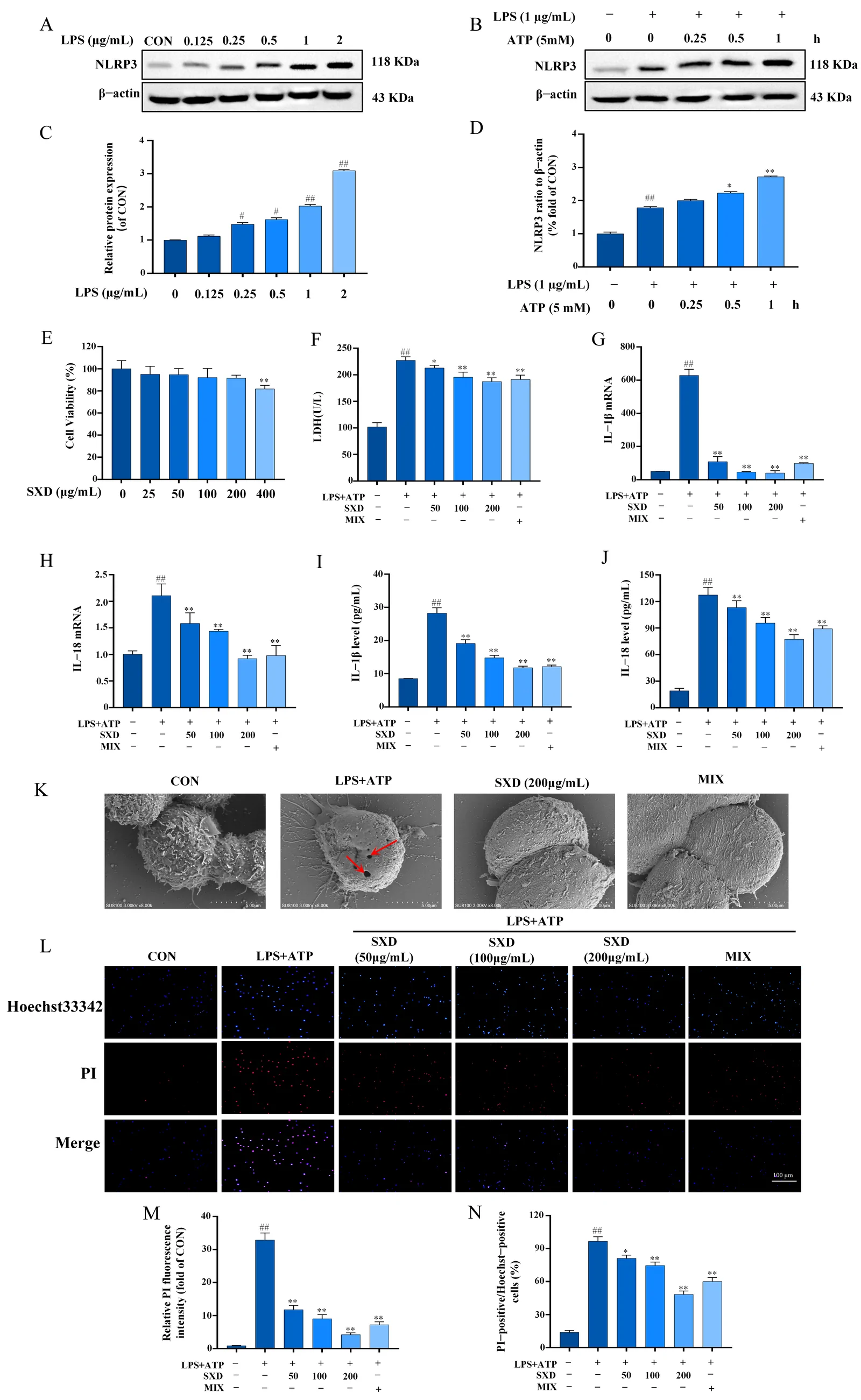
SXD inhibits pyroptosis in LPS/ATP-induced RAW264.7 macrophages. (**A**–**D**) Optimization of LPS concentration and ATP stimulation duration for modeling. (**E**) Effect of SXD on cell viability. (**F**) LDH release assay. (**G**–**J**) Effects of SXD on the levels and mRNA expression of IL-1β and IL-18. (**K**) SEM images of cells from each treatment group; red arrows indicate pore-like membrane discontinuities in the LPS/ATP group. (**L**–**N**) Hoechst 33342/PI staining, relative PI fluorescence intensity, and the percentage of PI-positive/Hoechst-positive cells. Data are presented as the mean ± SD from three independent biological experiments. * *p* < 0.05 and ** *p* < 0.01 versus the LPS/ATP group; # *p* < 0.05 and ## *p* < 0.01 versus the CON group.

To establish a stable in vitro model of inflammation and pyroptosis, the LPS/ATP induction conditions were optimized by Western blot analysis (Figure 3A–D). The final conditions were determined as stimulation with 1 μg/mL LPS for 12 h as the priming signal, followed by 5 mM ATP treatment for 1 h as the activation signal. As shown in Figure 3E, the selected noncytotoxic concentration range of SXD was 50–200 μg/mL.

To preliminarily evaluate the contribution of the selected constituents to the cellular effects of SXD, the four selected components were combined to form a mixture (MIX), which was used as a new comparison group in subsequent cell experiments [22,23]. The composition of MIX was determined based on the concentration of SXD (200 μg/mL) used in the high-dose group in cell experiments, and the contents of the four components were calculated accordingly. Taking kaempferol as an example, its concentration was calculated as 0.338% × 200 μg/mL × 1000/286.236 g/mol = 2.36 μM. Based on similar calculations, the final concentrations of the four components in MIX were determined as follows: kaempferol (2.36 μM), gentiopicroside (6.83 μM), chebulagic acid (0.92 μM), and berberine (2.95 μM). Unlike the initial single-compound screening performed at 20 μM, the MIX was prepared according to the measured content of each constituent in 200 μg/mL SXD and was not intended to represent an efficacy-equivalent dose.

Pyroptosis is characterized by loss of cell membrane integrity and subsequent release of LDH. In addition, activation of Caspase-1 promotes the cleavage and secretion of key inflammatory cytokines, including IL-1β and IL-18 [24]. Compared with the model group, both SXD and MIX significantly suppressed LDH release (Figure 3F) (*p* < 0.01) and reduced IL-1β and IL-18 transcription and secretion (Figure 3G–J) (*p* < 0.01). These findings suggest that SXD inhibited pyroptosis-related inflammatory responses, while MIX showed a partial inhibitory effect under the same in vitro conditions.

Cell-surface morphology was further examined by scanning electron microscopy (SEM; Figure 3K), together with Hoechst 33342/PI double staining (Figure 3L–N). LPS/ATP-treated cells displayed marked surface disruption and pore-like membrane discontinuities, whereas SXD- and MIX-treated cells showed comparatively preserved surfaces. Because SEM does not visualize intracellular ultrastructure, chromatin condensation and organelle swelling were not evaluated. In the Hoechst 33342/PI assay, LPS/ATP increased relative PI fluorescence and the percentage of PI-positive/Hoechst-positive cells, whereas both readouts were lower after SXD or MIX treatment.

### 2.5. SXD and MIX Mitigate DNA Damage in LPS/ATP-Induced RAW264.7 Cells

**Figure 4:**
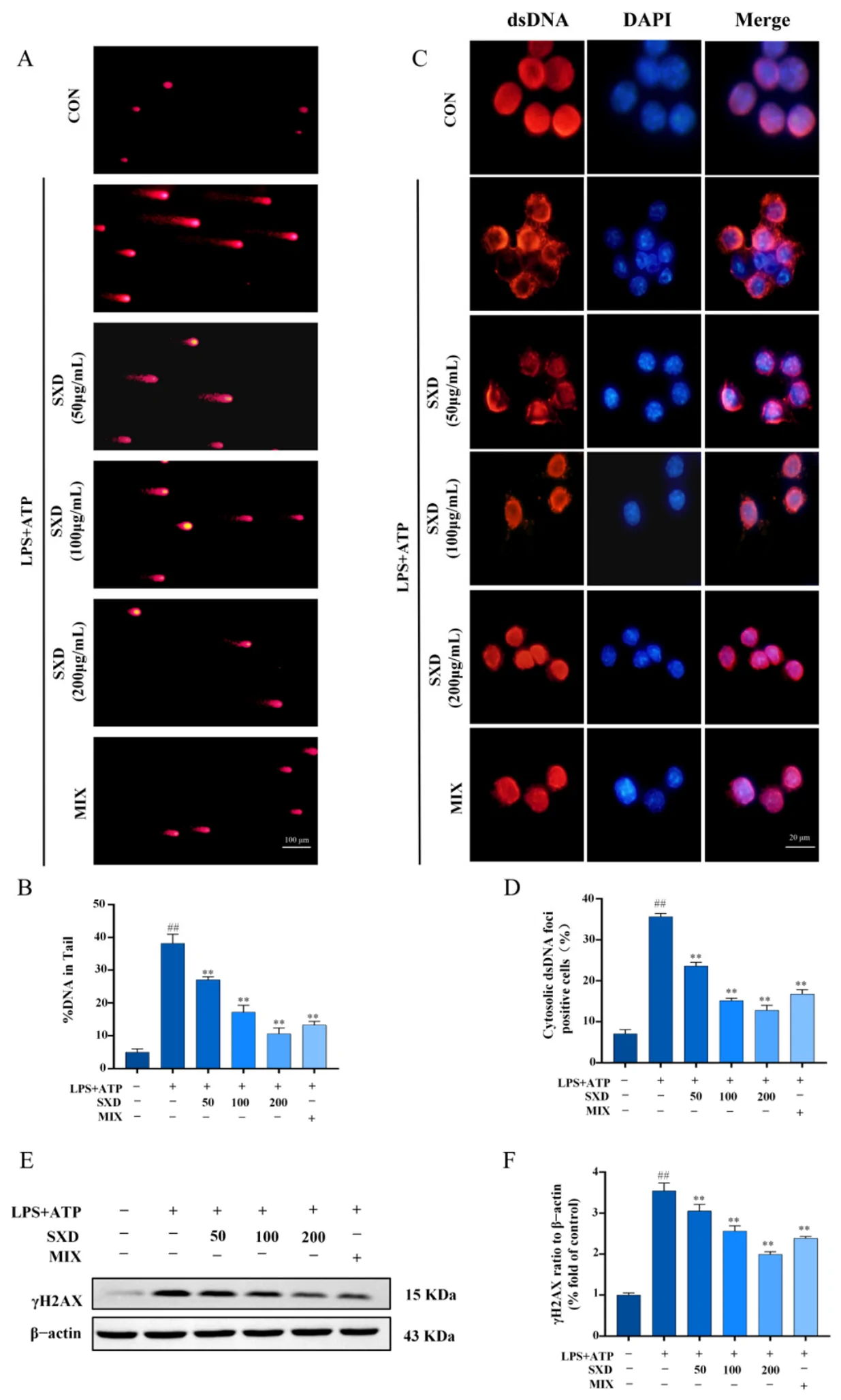
SXD and MIX attenuate DNA-damage-associated changes in RAW264.7 cells. (**A**,**B**) Representative alkaline comet images of DNA damage and quantification of the percentage of tail DNA (scale bar = 100 μm). (**C**,**D**) Representative immunofluorescence images of cytosolic dsDNA foci and quantitative analysis (scale bar = 20 μm). (**E**,**F**) Western blot assay and quantification of γH2AX protein levels. Data are presented as the mean ± SD from three independent biological experiments. ** *p* < 0.01 versus the LPS/ATP group; ## *p* < 0.01 versus the CON group.

The protective effects of SXD on DNA damage in RAW264.7 macrophages were evaluated using alkaline comet assay, Western blot, and immunofluorescence analyses. As shown in Figure 4A,B, LPS/ATP stimulation markedly increased comet tail length in RAW264.7 cells, indicating aggravated DNA fragmentation (*p* < 0.01). In contrast, treatment with SXD and MIX significantly shortened comet tail length and reduced DNA migration (*p* < 0.01), suggesting effective attenuation of DNA damage and maintenance of genomic stability. Consistently, LPS/ATP stimulation significantly upregulated the expression of the DNA double-strand break marker γH2AX, whereas SXD and MIX treatment markedly decreased γH2AX levels (Figure 4E,F; *p* < 0.01).

Cytosolic dsDNA is recognized as a critical upstream signal for cGAS/STING pathway activation [25]. Immunofluorescence analysis (Figure 4C,D) revealed that LPS/ATP stimulation induced the translocation of dsDNA (red fluorescence) from the nucleus to the cytoplasm, indicating nuclear DNA leakage and abnormal cytosolic accumulation. In contrast, SXD and MIX treatment significantly reduced cytosolic dsDNA signals (*p* < 0.01) and restored the predominantly nuclear localization. Taken together, SXD effectively alleviated LPS/ATP-induced DNA damage in RAW264.7 macrophages, thereby providing an important upstream mechanistic basis for the subsequent inhibition of cGAS/STING-mediated inflammatory responses.

### 2.6. Effects of SXD and MIX on cGAS/STING-Associated Signaling and Pyroptosis in Pol β-Knockdown RAW264.7 Cells

**Figure 5:**
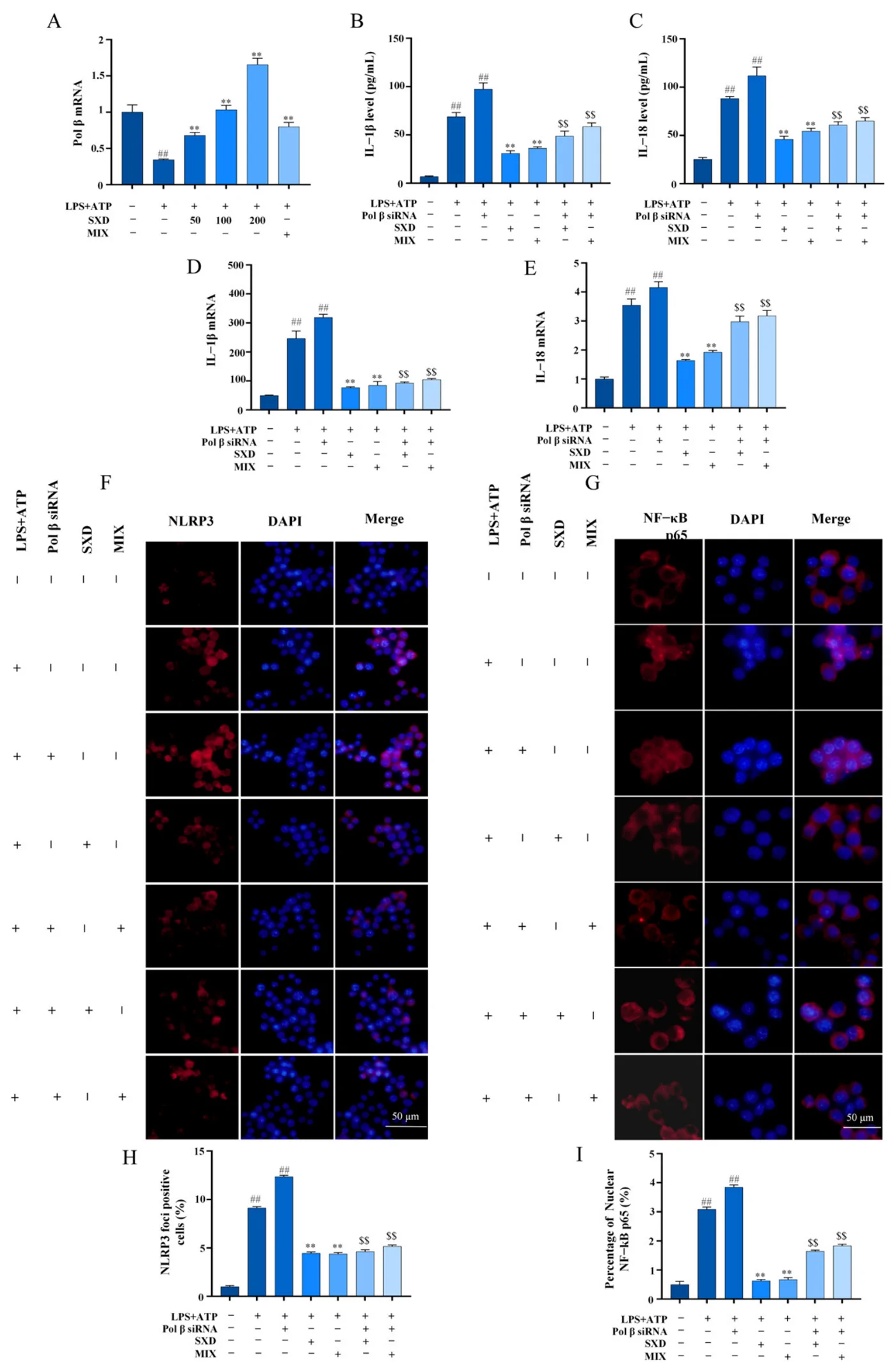
SXD attenuates macrophage pyroptosis under Pol β-knockdown. (**A**) RT-qPCR analysis of Pol β mRNA expression. (**B**–**E**) Effects of SXD and MIX on pyroptosis-associated cytokine expression and secretion. (**F**–**I**) Representative immunofluorescence images of NLRP3 staining and NF-κB p65 nuclear localization and quantitative analysis (scale bar = 50 μm). Data are presented as the mean ± SD from three independent biological experiments. ** *p* < 0.01 versus the LPS/ATP group; ## *p* < 0.01 versus the CON group; $$ *p* < 0.01 versus the LPS/ATP/Pol β siRNA group.

RT-qPCR analysis (Figure 5A) showed that SXD significantly upregulated Pol β mRNA expression in RAW264.7 macrophages (*p* < 0.01). To further investigate its role, Pol β was knocked down using siRNA (Figure S5). Following knockdown, IL-1β and IL-18 levels were significantly increased compared with the LPS/ATP group (Figure 5B–E) (*p* < 0.01). Although SXD and MIX still reduced these cytokines after Pol β knockdown, their effects were attenuated, which supported partial involvement of Pol β.

As shown in Figure 5F–I, LPS/ATP stimulation induced marked nuclear translocation of p65, whereas Pol β knockdown further enhanced both p65 nuclear translocation and phosphorylation levels (*p* < 0.01). Meanwhile, the fluorescence intensity of NLRP3 was also significantly increased compared with the LPS/ATP group (*p* < 0.01). These results suggest that Pol β knockdown promotes inflammatory signaling and inflammasome activation.

**Figure 6:**
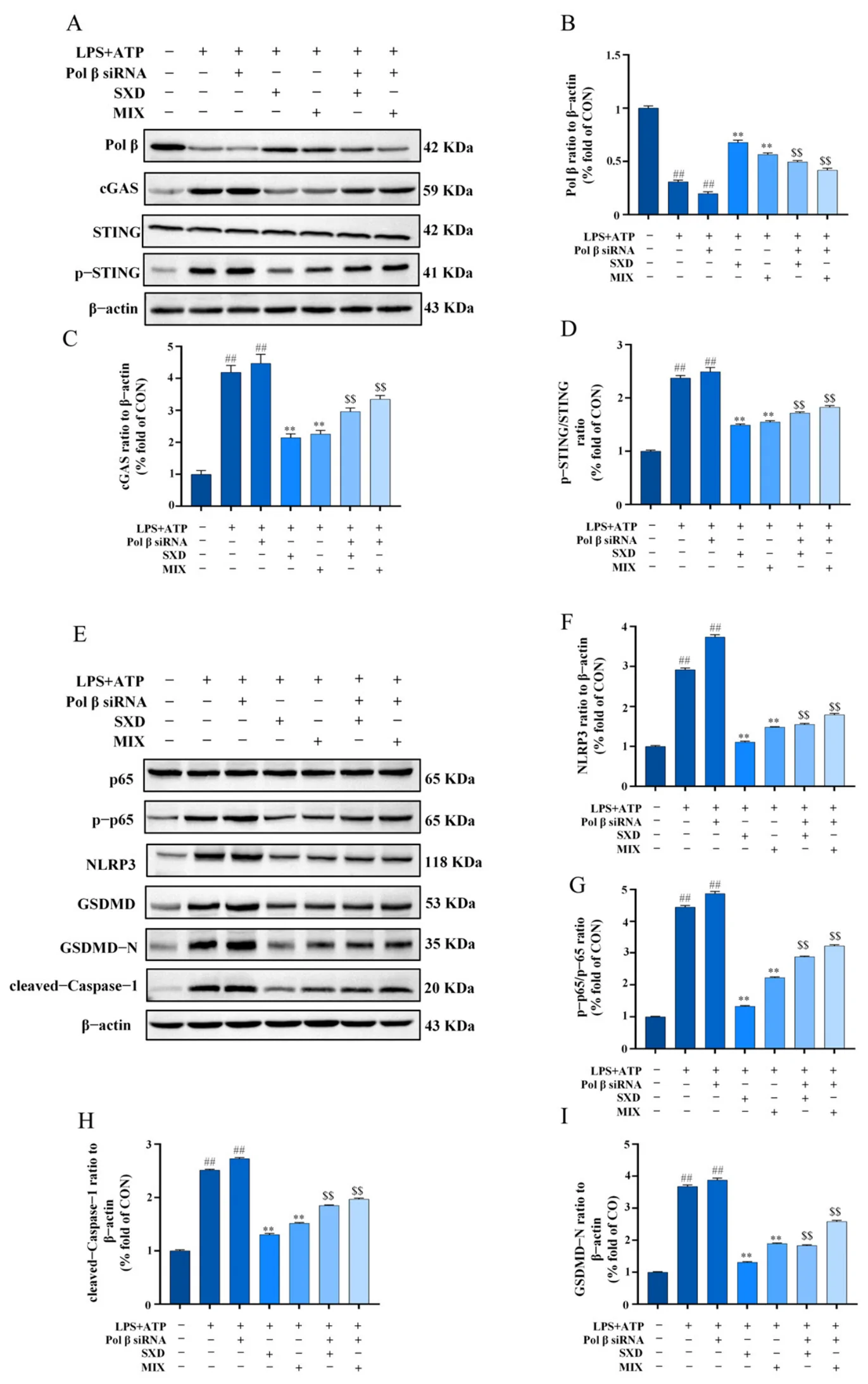
SXD attenuates cGAS/STING/NF-κB/NLRP3 pathway activation under Pol β-knockdown. (**A**–**D**) Western blot assay and the statistical data of Pol β, cGAS, p-STING, STING. (**E**–**I**) Western blot assay and the statistical data of p-p65, NLRP3, GSDMD-N, GSDMD and cleaved-Caspase-1 proteins. Data are presented as the mean ± SD from three independent biological experiments. ** *p* < 0.01 versus the LPS/ATP group; ## *p* < 0.01 versus the CON group; $$ *p* < 0.01 versus the LPS/ATP/Pol β siRNA group.

Western blot analysis (Figure 6A–I) further confirmed these results. Under Pol β knockdown conditions, the expression levels of cGAS, p-STING, p-p65, NLRP3, GSDMD-N, and cleaved-Caspase-1 were further elevated in LPS/ATP-induced RAW264.7 cells (*p* < 0.01), accompanied by a marked reduction in Pol β protein levels (*p* < 0.01). Notably, SXD and MIX treatment modulated the expression of these proteins to varying degrees (*p* < 0.01), exhibiting a certain dose-dependent trend.

Overall, Pol β knockdown exacerbated inflammation and pyroptosis. SXD, and to a lesser extent MIX, partially attenuated these alterations. The effects were weakened but not abolished after Pol β knockdown, supporting partial involvement of Pol β-associated processes in the cellular response.

### 2.7. Therapeutic Efficacy of SXD in CIA Rats

As shown in Figure S6B–F, the ankle joint diameter, paw thickness, and arthritis index (AI) in CIA rats were significantly increased on day 21 (*p* < 0.01). By day 30, treatment with H-SXD and MTX markedly reduced paw thickness and AI (*p* < 0.05 or *p* < 0.01). In addition, these pathological parameters were significantly improved in the L-SXD group on day 39, suggesting that SXD effectively attenuates arthritis progression in CIA rats.

**Figure 7:**
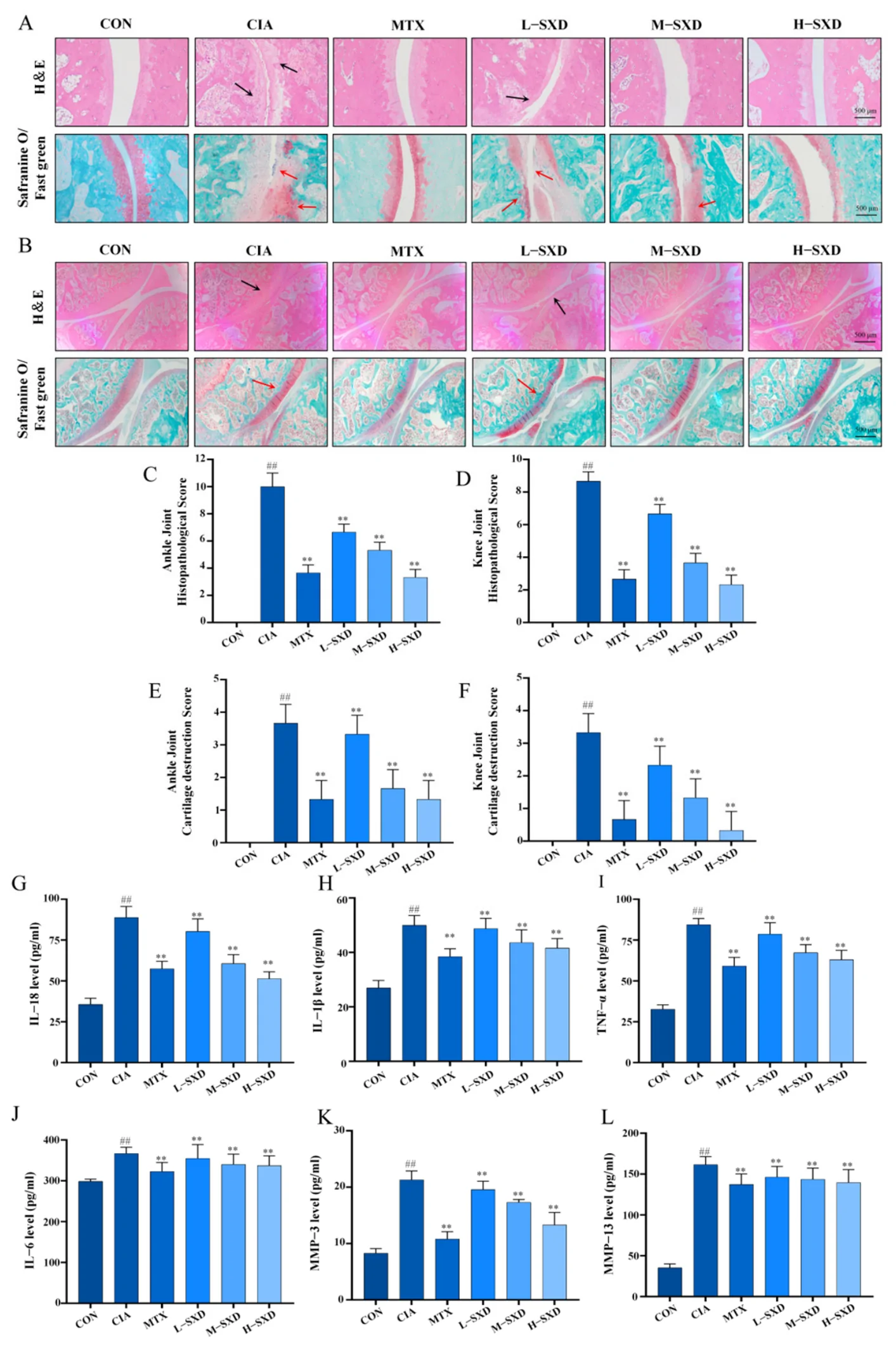
SXD attenuates inflammation and cartilage erosion in CIA rats. (**A**,**B**) Representative H&E- and Safranin O/Fast Green-stained sections of ankle and knee joints from CIA rats after the indicated treatments (n = 3 animals per group; scale bar = 500 μm). (**C**–**F**) Histopathological scores for synovial hyperplasia, inflammatory infiltration, and cartilage destruction. Black arrows indicate inflammatory infiltration and synovial hyperplasia, whereas red arrows indicate bone degradation and cartilage erosion. (**G**–**L**) Serum levels of TNF-α, IL-1β, IL-6, IL-18, MMP-3, and MMP-13 (n = 6 animals per group). Data are presented as mean ± SD. ** *p* < 0.01 versus the CIA group; ## *p* < 0.01 versus the CON group.

H&E and Safranin O/Fast Green staining of ankle joint sections (Figure 7A–F) showed that the CIA group exhibited significant synovial proliferation, inflammatory infiltration, and cartilage damage. SXD treatment significantly attenuated inflammatory infiltration and synovial hyperplasia in both ankle and knee joint tissues (*p* < 0.05 or *p* < 0.01). Furthermore, SXD significantly reduced the elevated serum levels of inflammatory cytokines, including TNF-α, IL-1β, IL-6, IL-18, as well as MMP-3 and MMP-13 in CIA rats (Figure 7G–L) (*p* < 0.01). These results indicate that oral administration of SXD effectively ameliorates joint lesions and inflammatory responses in CIA rats.

### 2.8. SXD Ameliorates DNA Damage and Related Pathway Activation in CIA Rats

Network pharmacology and docking results guided further investigation into the in vivo mechanisms of SXD in RA. The cGAS/STING pathway is a key cytosolic DNA-sensing pathway that triggers innate immune responses, while the generation of abnormal DNA is closely associated with the cell’s DNA repair capacity [25]. Pol β is a key BER enzyme involved in maintaining genomic stability [26].

**Figure 8:**
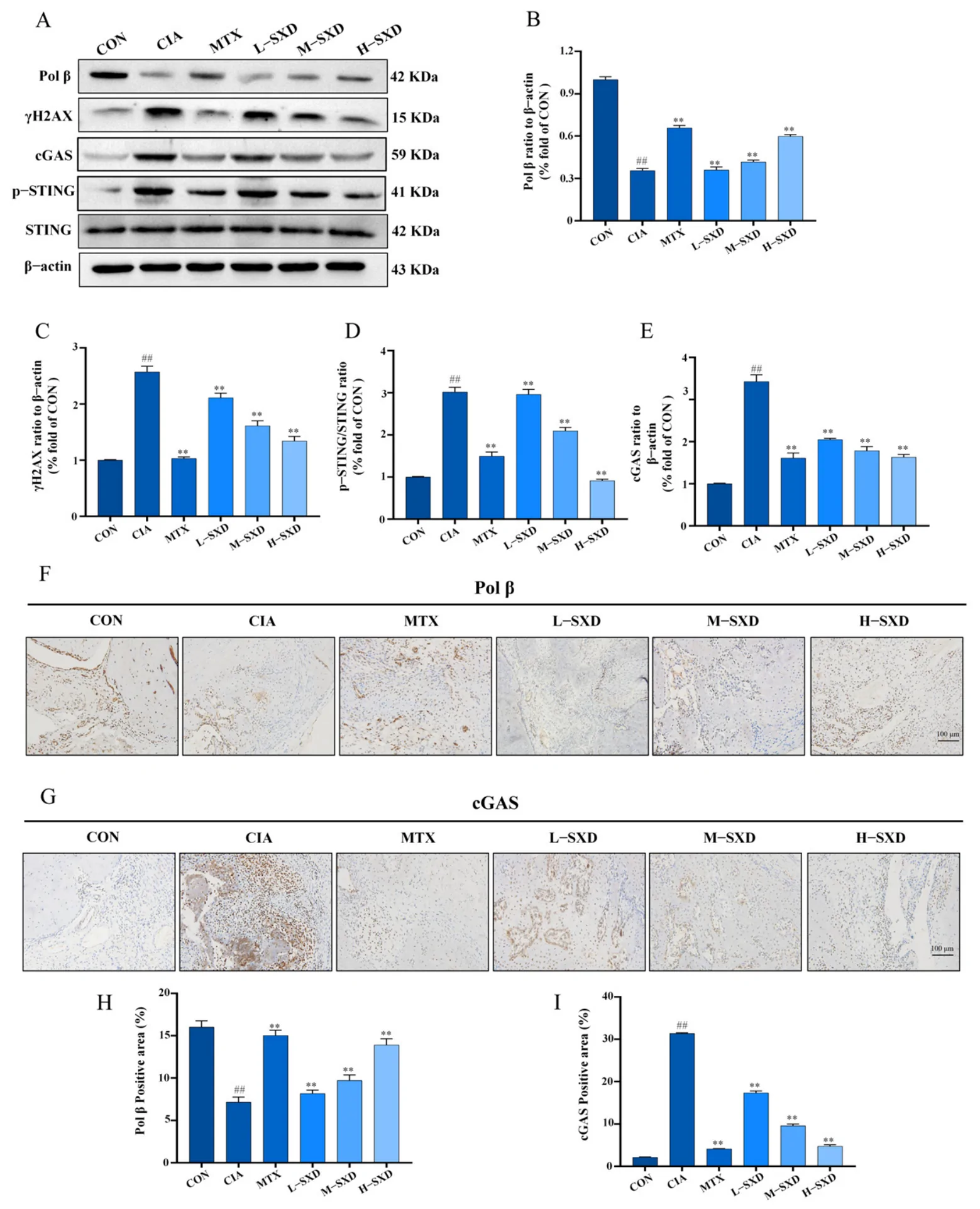
Effects of SXD on Pol β expression and cGAS/STING-related proteins in CIA rats. (**A**–**E**) Western blot analysis and quantification of Pol β, γH2AX, cGAS, and p-STING proteins (n = 3 animals per group). (**F**–**I**) IHC analysis of Pol β and cGAS expression in ankle joints (n = 3 animals per group; scale bar = 100 μm). Data are presented as mean ± SD. ** *p* < 0.01 versus the CIA group; ## *p* < 0.01 versus the CON group.

As shown in Figure 8A–E, CIA rats showed reduced Pol β expression together with altered cGAS/STING-related protein levels (*p* < 0.01). Oral administration of SXD markedly increased the protein level of Pol β (*p* < 0.01) while significantly suppressing the expression of cGAS and STING, which was consistent with the results of immunohistochemistry (Figure 8F–I) (*p* < 0.01). Meanwhile, the expression of γH2AX, a well-established marker of DNA double-strand breaks, was markedly increased in the CIA group (*p* < 0.01), indicating severe genomic damage. In contrast, SXD treatment significantly reduced γH2AX expression in a dose-dependent manner (*p* < 0.01).

### 2.9. SXD Inhibits Pyroptosis-Related Signaling Pathways in CIA Rats

**Figure 9:**
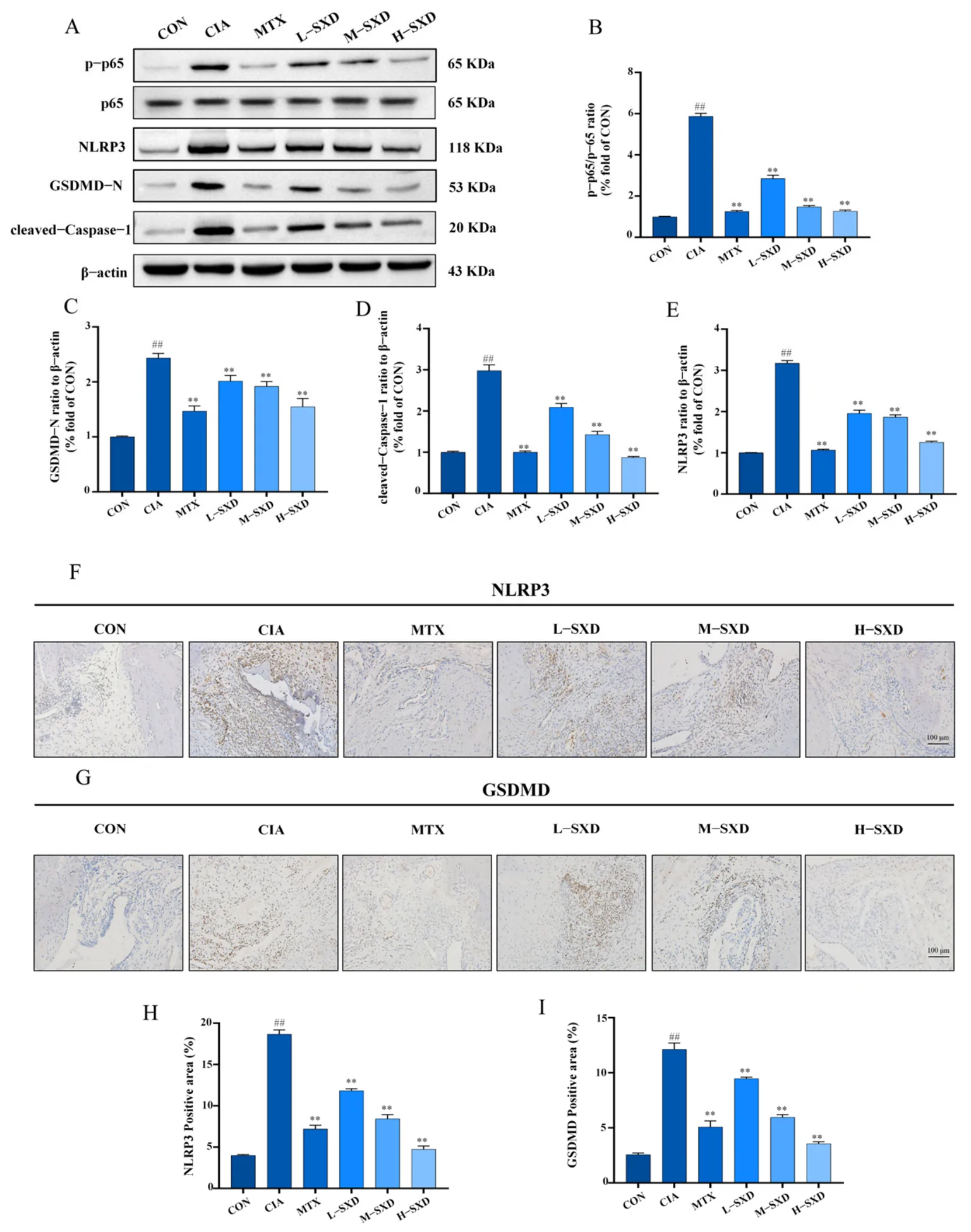
SXD ameliorates pyroptosis-related signaling pathways in CIA rats. (**A**–**E**) Western blot assay and the statistical data of p-p65, NLRP3, GSDMD-N and cleaved-Caspase-1 proteins (n = 3 animals per group). (**F**–**I**) IHC analysis of NLRP3 and GSDMD expression in ankle joints (n = 3 animals per group; scale bar = 100 μm). Data are presented as the mean ± SD. ** *p* < 0.01 versus the CIA group; ## *p* < 0.01 versus the CON group.

As shown in Figure 9A–E, Western blot analysis demonstrated that compared with controls, CIA rats exhibited significant upregulation of p-p65, NLRP3, GSDMD-N, and cleaved Caspase-1 (*p* < 0.01). These proteins are key mediators involved in NF-κB pathway activation and NLRP3 inflammasome-driven pyroptosis [24]. SXD treatment effectively reduced the expression levels of these proteins (*p* < 0.01). Furthermore, the immunohistochemical staining results were consistent with those obtained from Western blot analysis, further confirming the inhibitory effects of SXD on pyroptosis-related proteins (Figure 9F–I).

Taken together, SXD attenuated DNA damage by upregulating Pol β, thereby blocking the cGAS/STING pathway and reducing NF-κB/NLRP3-mediated pyroptosis in CIA rats. These findings preliminarily demonstrate that SXD exerts anti-RA effects through modulation of the Pol β/cGAS/STING/NF-κB/NLRP3 axis.

## 3. Discussion

RA is a multifactorial chronic autoimmune disorder involving inflammation, immune imbalance, and progressive tissue injury. Therefore, therapeutic strategies targeting multiple pathways and molecular targets are considered more effective [27]. As a classical Tibetan medicinal formula for the treatment of RA, SXD has been extensively investigated in our previous studies and has demonstrated significant anti-RA efficacy. Our previous study showed that SXD alleviates joint swelling and synovial inflammation by inhibiting the JAK2/STAT3 and NF-κB signaling pathways [20]. Furthermore, recent studies have revealed that SXD can ameliorate RA by modulating the gut microbiota–SCFA metabolism–immune homeostasis axis [21]. Collectively, these findings indicate that SXD exerts therapeutic effects against RA through the regulation of multiple molecular targets and signaling pathways. In contrast, the present study focuses on the relationships among DNA damage, Pol β expression, cytosolic dsDNA accumulation, cGAS/STING and NF-κB signaling, NLRP3 inflammasome activation, and pyroptosis. In the present study, network pharmacology and molecular docking were combined with UHPLC-Q-Orbitrap HRMS analysis of serum-absorbed constituents and in vivo and in vitro experiments to characterize candidate constituents and examine DNA-damage- and pyroptosis-associated responses.

Network pharmacology was further used to associate serum-absorbed constituents with RA targets, leading to the construction of a “compound-target-pathway” network. These targets were predominantly enriched in innate immunity- and pyroptosis-associated inflammatory pathways, including NF-κB, NLRP3, and cGAS/STING. Although network pharmacology identified several RA-related pathways, the cGAS/STING–NF-κB–NLRP3 axis was prioritized because it provided a coherent and experimentally testable link among Pol β-associated DNA damage, cytosolic dsDNA sensing, and macrophage pyroptosis. Other enriched pathways were beyond the scope of the present study and require independent validation. Accumulating evidence indicates that the accumulation of DNA damage and defects in DNA repair are critical driving factors in autoimmune disease pathogenesis, including RA [28,29,30]. Pol β is critically involved in the BER pathway and helps maintain genomic stability by repairing oxidative and alkylation-related base lesions as well as single-strand breaks [31]. Pol β deficiency induces DNA damage accumulation and cytosolic dsDNA release, thereby activating cGAS/STING signaling and downstream TBK1/IKK-dependent IRF3 and NF-κB signaling, which promotes the expression of pro-inflammatory cytokines [32]. It has been reported that Pol β knockout in mice results in early embryonic lethality [7], underscoring its essential role in genomic maintenance. Notably, beyond Pol β, alterations in other DNA damage sensors or repair proteins can also induce DNA damage accumulation and nuclear DNA leakage into the cytoplasm [33].

Cytosolic self-DNA can serve as a major trigger of cGAS/STING signaling [34]. Although cGAS exhibits limited sequence specificity, it is highly sensitive to the spatial context of double-stranded DNA (dsDNA) and can robustly respond to cytosolic DNA derived from nuclear or mitochondrial damage [35]. In addition to cGAS, multiple other molecules have been identified as DNA sensors [36,37]. cGAS and absent in melanoma 2 (AIM2) are important dsDNA sensors involved in macrophage pyroptosis. Through Caspase-1 activation, both promote the processing and release of IL-1β and IL-18; however, AIM2 primarily functions in host defense against DNA viruses and certain cytosolic bacterial pathogens [38]. Therefore, the dysregulated activation of these DNA sensors may contribute to inflammatory amplification and macrophage pyroptosis in RA pathogenesis.

Overall, our data support that SXD protects against RA by attenuating DNA damage, modulating innate immune regulation, and suppressing pyroptosis-related responses. In vitro, LPS/ATP stimulation induced pronounced DNA damage in RAW264.7 macrophages, as evidenced by increased γH2AX expression, enhanced comet tail formation, and cytosolic leakage of nuclear dsDNA. Consistently, elevated γH2AX levels were also observed in joint tissues of CIA rats, suggesting the presence of DNA damage in vivo. Notably, SXD and MIX increased Pol β expression in RAW264.7 cells. These changes were accompanied by reduced γH2AX expression, shorter comet tails, and decreased cytosolic dsDNA accumulation, indicating attenuation of DNA-damage-associated alterations. In CIA rats, SXD also restored the reduced Pol β expression in joint tissues and decreased γH2AX expression, together with reductions in cGAS expression and STING phosphorylation. These parallel changes suggest that increased Pol β expression may contribute to the maintenance of genomic stability and thereby limit the accumulation of damage-derived cytosolic DNA and the associated cGAS/STING signaling response. However, because BER activity, repair kinetics, and Pol β enzymatic activity were not directly measured, the present findings do not establish that SXD directly enhances Pol β-mediated DNA repair. In addition, cGAMP production, STING trafficking, TBK1 phosphorylation, and IRF3 activation were not measured. The results therefore support cGAS/STING-associated signaling changes rather than a complete functional characterization of canonical pathway activation.

Mechanistically, knockdown of Pol β markedly enhanced activation of the cGAS/STING pathway and phosphorylation of p65 in RAW264.7 cells, accompanied by overproduction of pro-inflammatory cytokines. Although SXD and MIX were still able to partially attenuate these alterations, their protective effects were significantly diminished, indicating that Pol β plays a pivotal regulatory role in this signaling cascade and may serve as a critical node in maintaining cellular immune homeostasis. Meanwhile, the partial efficacy of SXD retained after Pol β knockdown suggests the existence of additional Pol β-independent mechanisms. Further investigation demonstrated that SXD and MIX significantly inhibited NF-κB activation, GSDMD-N-driven membrane pore formation, and the secretion of IL-1β and IL-18, indicating that they suppress the amplification of inflammation triggered by DNA damage. Consistently, SXD also exerted pronounced inhibitory effects on inflammatory cytokine production and pyroptosis pathway activation in CIA rats. Under conditions of Pol β knockdown, the expression of pyroptosis-related proteins was further elevated, whereas SXD and MIX could still partially reverse this trend, suggesting that their anti-pyroptotic effects are at least partly associated with Pol β expression.

Meanwhile, four selected constituents detected in medicated serum were identified from SXD, namely chebulagic acid, kaempferol, gentiopicroside, and berberine. Notably, these constituents have each been independently reported to exert anti-inflammatory and joint-protective effects in RA or RA-related models. For instance, kaempferol and berberine exhibit protective effects in CIA rats by suppressing synovial inflammation and limiting bone erosion [39,40]; gentiopicroside improves experimental arthritis by suppressing NF-κB activation and reducing oxidative stress levels [41]; and chebulagic acid exhibits potent inhibitory effects on inflammatory cell infiltration and cytokine release, while improving arthritic symptoms and regulating immune responses [42]. To preliminarily assess the possible contribution of these constituents, we adopted a strategy based on the SXD concentration used in the high-concentration cell treatment group. These four constituents were quantitatively determined by HPLC and subsequently combined into a defined MIX for in vitro administration. It should be noted that the MIX used in this study was constructed as a content-based in vitro combination of selected constituents, rather than an efficacy-equivalent substitute for SXD. Although MIX partially reproduced the inhibitory effects of SXD on inflammatory cytokine release, DNA damage, and pyroptosis-related signaling in RAW264.7 cells, its activity was evaluated only in vitro and was not validated in CIA rats. Therefore, the present findings only suggest that these constituents may contribute to the cellular pharmacological effects of SXD. Further pharmacokinetic studies and in vivo comparisons are required to determine whether these constituents can serve as definitive quality markers or simplified substitutes for SXD. Moreover, this procedure did not quantify the contribution of each compound to the overall activity of SXD, and no formal interaction analysis was performed. Future studies using formal interaction designs will evaluate their potential coordinated effects.

Apart from these issues, several additional limitations of the present study should also be acknowledged. First, the specific constituents within SXD that directly target Pol β remain unclear. In addition, only the prototype compounds present in rat serum were identified, whereas the metabolic profiles of these constituents were not characterized. Although MIX partially reproduced some in vitro effects of H-SXD, its activity was weaker than that of H-SXD in certain cellular parameters, suggesting that additional constituents in the original formula may exert synergistic or protective effects. These issues warrant further investigation in future studies. Finally, future studies should further clarify the mechanisms underlying the effects of SXD by evaluating Pol β activity, DNA repair capacity, and canonical cGAS/STING signaling. Validation in additional models, including primary macrophages, fibroblast-like synoviocytes, and human RA-derived cells, should be conducted.

## 4. Materials and Methods

### 4.1. Chemicals and Reagents

The selection of reference standards was based on our previous laboratory studies. Methanol and acetonitrile of HPLC grade were sourced from Merck KGaA (Darmstadt, Germany).

**Figure 10:**
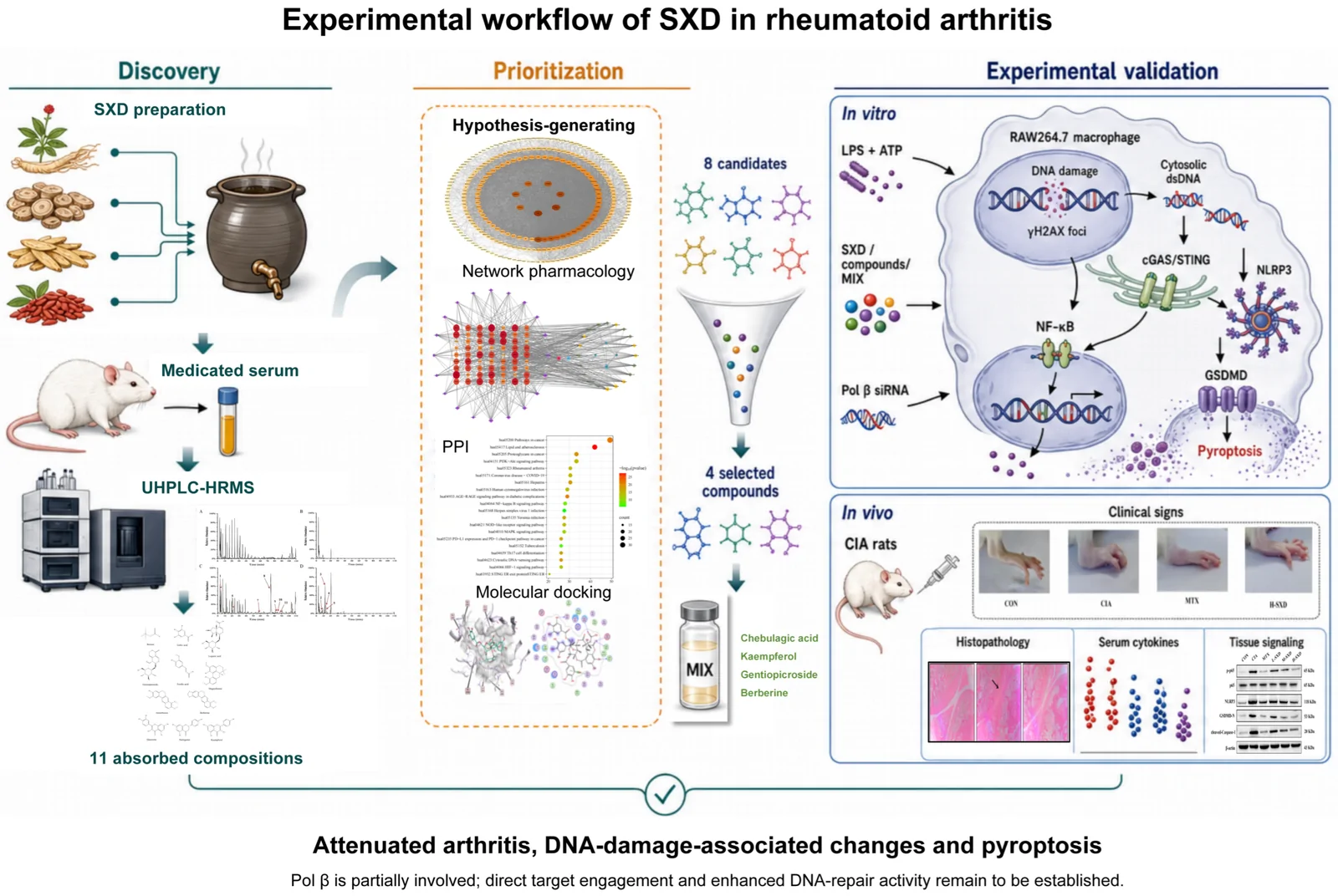
Methodological flowchart.

RAW264.7 cells (CL-0190), a murine macrophage cell line, were sourced from Procell Life Science & Technology Co., Ltd. (Wuhan, China) and cultured in a complete medium (CM-0190; Procell, Wuhan, China). ELISA kits for IL-1β (EK0394), IL-6 (EK0411), IL-18 (EK0868), TNF-α (EK0527), MMP-3 (EK0465) and MMP-13 (EK0466) were obtained from Boster Biological Technology Co., Ltd. (Wuhan, China). LDH assay kit (A020-2-2) was obtained from Nanjing Jiancheng Bioengineering Institute (Nanjing, China). Sigma-Aldrich (St. Louis, MO, USA) supplied LPS (L2880) and ATP (A7699), whereas MTT (M8180) and the BCA protein assay kit (PC0020) were provided by Beijing Solarbio Science & Technology Co., Ltd. (Beijing, China). Primary antibodies, including anti-Pol β (AF7483), anti-cGAS (AF6030), anti-STING (AF7895), anti-phospho STING (AF8067), anti-p65 (AF5006), anti-phospho p65 (AF2006), anti-NLRP3 (AF2158), anti-GSDMD (AF4012), anti-GSDMD-N (DF12275), anti-γH2AX (AF2288) and anti-β actin (AF7018), were purchased from Affinity Biosciences (Cincinnati, OH, USA). Methotrexate (MTX, H31020644) was obtained from SPH Sine Pharmaceutical Laboratories Co., Ltd (Shanghai, China). Freund’s incomplete adjuvant (F5506) was sourced from Sigma-Aldrich (St. Louis, MO, USA), and bovine type II collagen (20021) was purchased from Chondrex, Inc. (Woodinville, WA, USA). Antibody identity and application suitability were checked against the manufacturers’ datasheets, and immunoblot signals were evaluated at the expected molecular masses. No independent genetic validation of individual antibodies was performed in this study. The experimental design and workflow are summarized in Figure 10.

### 4.2. Preparation and Chemical Composition Analysis of SXD

SXD sample and standard preparation, along with chromatographic procedures, were performed according to methods described in our earlier study [20]. For chromatographic analysis, both samples and reference compounds were dissolved in methanol (HPLC grade) and filtered before injection. An Agilent 1260 HPLC system (Agilent Technologies, Santa Clara, CA, USA) coupled with a Hypersil™ BDS C18 column (Thermo Fisher Scientific, Waltham, MA, USA) was employed for separation. The mobile phase consisted of 1% phosphoric acid in water (A) and acetonitrile (B), and the detailed gradient program is listed in Table S1.

### 4.3. Analysis of SXD by Network Pharmacology

#### 4.3.1. Drug Target and Disease Target Acquisition

Candidate active constituents of SXD were identified by integrating chromatographic data with information from TCMSP (https://www.tcmsp-e.com/load_intro.php) (accessed on 6 December 2025) and BATMAN-TCM (http://bionet.ncpsb.org.cn/batman-tcm/, accessed on 6 December 2025). Twenty major constituents were further screened using SwissADME. SwissTargetPrediction (http://www.swisstargetprediction.ch/) was used to predict compound-related targets, whereas RA-related targets were retrieved from DrugBank (https://go.drugbank.com/), OMIM (https://www.omim.org/), and GeneCards (https://www.genecards.org/; relevance score > 5). Overlapping compound- and disease-related targets were retained as predicted targets of SXD in RA.

#### 4.3.2. Protein–Protein Interaction (PPI) Network Analysis Combined with GO and KEGG Enrichment Analysis

To construct the PPI network, the overlapping targets were submitted to the STRING (https://string-db.org/) platform with a minimum confidence score of 0.9. Hub targets were identified through topological analysis in Cytoscape 3.9.1 (https://cytoscape.org/) using degree as the key parameter. Using DAVID (https://davidbioinformatics.nih.gov), GO functional annotation and KEGG pathway enrichment were performed with *p* < 0.05 set as the cutoff, and the 10 most significant GO terms and 20 KEGG pathways were visualized. Finally, to illustrate the multi-target regulatory effects of SXD, a “herb-component-target-pathway” network was constructed using Cytoscape.

#### 4.3.3. Molecular Docking

Based on the PPI network, eight candidate compounds were docked against Caspase-1 (1RWP), IL-1β (8C3U), STING (7SSM), NF-κB p50 (1NFK), NLRP3 NACHT (7ALV), IL-6 (1ALU), and TNF-α (2AZ5). Ligands and proteins were obtained from PubChem and the Protein Data Bank (https://www.rcsb.org/), respectively. Docking was performed using AutoDock 4.2.6 (The Scripps Research Institute, La Jolla, CA, USA) (https://autodock.scripps.edu/) and AutoDockTools 1.5.6. Receptors were prepared by removing water, unrelated heteroatoms, and co-crystallized ligands and adding polar hydrogens and Kollman charges. Gasteiger charges and rotatable bonds were assigned to ligands. Proteins were treated as rigid and ligands as flexible. AutoGrid maps comprised 60 × 60 × 60 points at a 0.375 Å spacing and were centered on the known binding site or a predefined functional pocket. The Lamarckian genetic algorithm was run 100 times with a population size of 150, 2.5 × 10^6^ energy evaluations, and 27,000 generations. Poses were clustered at a 2.0 Å RMSD tolerance. Because protein-specific grid centers were not retained and native-ligand redocking was not performed, redocking RMSD values were unavailable. Docking scores were interpreted as hypothesis-generating predictions rather than evidence of direct binding or target engagement.

### 4.4. Method Validation and Fingerprint Profile Establishment

The HPLC method was validated for precision, repeatability, and accuracy. Precision was assessed by six repeated injections of one mixed standard solution, while repeatability was evaluated using six independently prepared SXD (S1) samples. Accuracy was determined by spike recovery tests with 1.0 g SXD. In addition, 20 batches of SXD were analyzed under the same conditions, and chromatograms were processed using the Chinese Medicine Chromatogram Fingerprint Similarity Evaluation System (2012 version) for fingerprint generation and similarity analysis.

### 4.5. Cell Experiments

#### 4.5.1. Cell Culture and Establishment of the Inflammation Model

Cells were cultured in DMEM supplemented with 10% fetal bovine serum and 1% penicillin–streptomycin at 37 °C in a humidified atmosphere containing 5% CO_2_. Pyroptosis was induced in RAW264.7 cells by priming with 1 μg/mL LPS for 12 h and subsequently activating with 5 mM ATP for 1 h.

#### 4.5.2. Cell Viability Assay

The MTT assay was employed to determine cell viability. RAW264.7 cells were plated in 96-well plates at a density of 1 × 10^5^ cells/mL and cultured for 12 h prior to treatment with different concentrations of SXD (50–200 μg/mL) for 12 h. After 4 h of incubation with MTT, the optical density was measured at 490 nm. The replicate structure followed that described at the end of Section 4.5.

#### 4.5.3. Cell Immunofluorescence (IF) Staining

Cells were allowed to adhere for 12 h and were then pretreated with SXD for an additional 12 h. The medium was then refreshed with drug-containing medium, followed by model induction as described above. Cells were fixed using 4% paraformaldehyde, washed with PBS, and permeabilized with 0.1% Triton X-100. Cells were incubated overnight at 4 °C with primary antibodies against dsDNA, NLRP3, and p65. Afterward, cells were treated with Alexa Fluor 488-labeled secondary antibody and stained with DAPI. Images were analyzed in ImageJ 1.54t (National Institutes of Health, Bethesda, MD, USA); the percentages of cells exhibiting cytosolic dsDNA foci, NLRP3-positive staining, or nuclear p65 localization were calculated relative to the total number of DAPI-positive cells. Measurements from multiple fields within each independent culture were averaged to obtain one biological-replicate value.

#### 4.5.4. Alkaline Comet Assay

After the indicated treatments, an alkaline comet assay was performed to assess DNA migration. Comet images were acquired by fluorescence microscopy, and DNA damage was quantified as the percentage of DNA in the comet tail. Measurements from multiple cells within each independent culture were averaged to obtain one biological-replicate value.

#### 4.5.5. Quantitative Real-Time PCR (qRT-PCR)

RNA from treated RAW264.7 cells was isolated with the TRIzol reagent according to standard procedures. Its concentration and purity were measured using a NanoDrop™ Lite spectrophotometer (Thermo Fisher Scientific, Waltham, MA, USA) spectrophotometer. After reverse transcription into cDNA, quantitative real-time PCR was conducted on a QuantStudio 1 platform (Applied Biosystems, Thermo Fisher Scientific, Waltham, MA, USA). Gene expression levels were normalized to β-actin and analyzed using the 2^−ΔΔCt^ method, with primer sequences provided in Table S2.

#### 4.5.6. Hoechst 33342/PI Double Staining

Morphological assessment of RAW264.7 cells was conducted using Hoechst 33342/PI staining. Cells were plated in six-well plates, treated as described, and sequentially stained with Hoechst 33342 and PI. Fluorescence images were captured under a microscope. Relative PI fluorescence intensity and the percentage of PI-positive/Hoechst-positive cells were quantified in Image J 1.54t. Measurements from multiple fields within each independent culture were averaged to obtain one biological-replicate value.

#### 4.5.7. Scanning Electron Microscopy

Cell-surface morphology was examined using an SU8100 field-emission scanning electron microscope (Hitachi High-Tech Corporation, Tokyo, Japan) at an accelerating voltage of 3.0 kV. The SEM images were used to assess surface integrity and pore-like membrane discontinuities; intracellular chromatin and organelle morphology were not evaluated from these images.

#### 4.5.8. Cell Transfection

Cells were seeded into 6-well plates. GP-transfect-Mate reagent was mixed with Pol β RNA oligo to form transfection complexes by incubating at room temperature for 15–20 min, which were used within 60 min without altering the mixing order. The complexes were then added to the cells for incubation, followed by detection of protein expression levels. The sequences of Pol β RNA oligo are listed in Table S3.

Unless otherwise specified, all cell-based experiments were independently conducted three times on three different days using separately cultured, seeded, and treated cells; each independent experiment was considered one biological replicate. For plate-based assays, three parallel wells per condition were treated as technical replicates and averaged to obtain one value for each biological replicate. For RT-qPCR, three technical PCR wells were averaged before ΔCt and ΔΔCt calculations. For microscopy and comet assays, measurements from multiple fields or cells within the same culture were averaged to generate one value per biological replicate. Technical replicates were not treated as independent observations.

### 4.6. Animal Experimentation

#### 4.6.1. Animals and Ethics

Male Sprague–Dawley (SD) rats (180–200 g) were sourced from the Qingdao Institute for Food and Drug Control (Qingdao, China; License No. SYXK (Lu) 20230410). The animals were acclimatized for seven days under standardized conditions (12-h light/dark cycle, 22 ± 2 °C and 55 ± 5% humidity) with ad libitum access to standard chow and water. The Animal Ethics Committee of Qingdao University of Science and Technology reviewed and authorized all experimental procedures (Approval No. QKDLL2022-2L, Approval date: 5 March 2024).

#### 4.6.2. Preparation of Medicated Serum

Thirty male SD rats were allocated to CON and SXD-treated groups (n = 15 per group). The SXD group received 19.845 g/kg/day of SXD (seven-fold the clinical equivalent dose) by oral gavage twice daily for 7 days. One hour after the final dose, rats were euthanized under anesthesia, and blood was collected to obtain medicated serum for subsequent analysis.

#### 4.6.3. Serum Sample Processing and Ingredient Testing

Pooled serum samples (200 μL) from each group were treated with an equal volume of methanol for protein precipitation. After vortexing for 3 min and centrifugation (15,000 rpm, 10 min, 4 °C), following repeated extraction, the supernatants were pooled and dried under nitrogen. The residue was redissolved in 100 μL of methanol and passed through a 0.22 μm organic membrane filter. A 2 μL volume of the filtrate was then injected into the UHPLC-Orbitrap Exploris™ 120-MS (Thermo Fisher Scientific, Waltham, MA, USA).

An Agilent ZORBAX SB-C18 column (Agilent Technologies, Santa Clara, CA, USA) (4.6 × 100 mm, 1.8 μm) was used at 0.3 mL/min with 0.1% formic acid in water (A) and acetonitrile (B). The gradient was 9–12% B at 0–10 min, 12–17% B at 10–30 min, 17–19% B at 30–55 min, 19–25% B at 55–70 min, 25–45% B at 70–100 min, 45–60% B at 100–105 min, and 60–95% B at 105–110 min. The column temperature was 40 °C, and the injection volume was 2 μL. Mass spectra were acquired in positive- and negative-ion electrospray modes over m/z 100–1500, with spray voltages of +3.5 kV and −3.0 kV, respectively, a sheath-gas setting of 35 L/h, and a capillary temperature of 350 °C.

#### 4.6.4. CIA Model Induction and Experimental Grouping

The collagen-induced arthritis (CIA) model was established using a protocol refined in our previous work [20]. Briefly, rats were immunized with a 1:1 emulsion of bovine type II collagen and incomplete Freund’s adjuvant (200 μL) on days 0 and 21. After successful CIA induction (arthritis index, AI ≥ 4), rats were stratified by body weight and allocated to six groups (n = 6 per group). Using body-surface-area conversion with a human-to-rat factor of 6.3, according to the method described by Reagan-Shaw et al. [43,44], the corresponding rat dose was 2.835 g crude herbs/kg/day, which was adopted as the middle CIA dose (M-SXD); the L-SXD (1.4175 g/kg) and H-SXD (5.67 g/kg) doses were set at 0.5 and 2.0 times the M-SXD dose, respectively. It should be noted that a higher dose of 19.845 g crude herbs/kg/day (seven times the clinically equivalent rat dose) was used only for the 7-day medicated-serum preparation protocol to facilitate detection of circulating prototype constituents by UHPLC–HRMS, and was not applied in this 4-week CIA efficacy experiment. Within each body-weight stratum, consecutively numbered rats were assigned according to a computer-generated random-number sequence produced with the RAND function in Microsoft Excel; one animal from each stratum was allocated to each group in ascending order of the generated random numbers.

SXD and saline were administered daily via oral gavage for four weeks, whereas MTX was given twice weekly. Clinical symptoms, including AI scores, paw thickness and ankle diameter, were monitored every three days. Twenty-four hours after the final administration, all rats were anesthetized for the systemic collection of serum, plasma, and joint specimens (knee and ankle) for downstream analysis.

#### 4.6.5. ELISA Analysis

The levels of TNF-α, IL-1β, IL-6, IL-18, MMP-3, and MMP-13 in rat serum and those of IL-1β and IL-18 in RAW264.7 cell culture supernatants were measured using the corresponding ELISA kits. LDH activity in cell supernatants was measured using the LDH assay kit described in Section 4.1.

#### 4.6.6. Histological and Immunohistochemical (IHC) Analysis

Paraffin-embedded ankle and knee joints were sectioned and stained with H&E and Safranin O/Fast Green to assess synovial hyperplasia and cartilage degradation. Sections were examined using a Provis AX70 light microscope (Olympus Corporation, Tokyo, Japan). RA severity was quantified using published histopathological and cartilage-destruction scoring methods [45,46]. For IHC, sections underwent deparaffinization, sodium-citrate antigen retrieval, endogenous peroxidase blocking with 3% H_2_O_2_, and blocking with 10% goat serum. Sections were incubated overnight at 4 °C with antibodies against cGAS, Pol β, NLRP3, and GSDMD. Immunoreactivity was visualized using biotinylated secondary antibodies and a diaminobenzidine kit. All slides were anonymized using coded identifiers, and histopathological scoring and IHC quantification were independently performed by two investigators blinded to treatment allocation; discrepant histopathological scores were resolved by joint re-examination and consensus, whereas the mean of the two independently obtained IHC measurements was used for statistical analysis.

### 4.7. Western Blotting Analysis

Proteins were isolated from rat ankle tissues and RAW264.7 cells with RIPA lysis buffer (HY-K1001, MCE, Monmouth Junction, NJ, USA) and quantified via a BCA assay kit (PC0020, Solarbio, Beijing, China). Protein samples of equal quantity were subjected to SDS-PAGE, followed by transfer to membranes. Following blocking, the membranes were incubated overnight at 4 °C with primary antibodies (1:1000) against Pol β, cGAS, STING, p-STING, p65, p-p65, NLRP3, GSDMD, GSDMD-N, γH2AX, and β-actin. Bands were detected using ECL and quantified by ImageJ-based densitometric analysis.

### 4.8. Statistical Analysis

Statistical analyses were performed using GraphPad Prism 9.5.1 (GraphPad Software, Boston, MA, USA). Data are presented as the mean ± standard deviation (SD). Normality was assessed using the Shapiro–Wilk test. Comparisons between exactly two independent groups, including validation of Pol β knockdown between the si-NC and si-Pol β groups, were performed using an unpaired two-tailed Student’s *t*-test. End-point assays involving three or more independent groups were analyzed using one-way ANOVA followed by Tukey’s multiple-comparisons test. Longitudinal measurements of body weight, paw thickness, ankle diameter, and arthritis index were analyzed using two-way repeated-measures ANOVA followed by Šídák’s multiple-comparisons test.

For cell-based assays, n = 3 represents three independent biological experiments conducted on different days; technical replicate wells, PCR reactions, microscopic fields, or individual cells were first averaged within each independent experiment. For animal experiments, each rat constituted one biological replicate. Clinical assessments and serum measurements used six rats per group (n = 6), whereas histopathological scoring, IHC quantification, and tissue Western blot analyses used three animals per group (n = 3), as specified in the figure legends. All tests were two-sided, and *p* < 0.05 was considered statistically significant.

## 5. Conclusions

SXD ameliorated clinical, histological, and inflammatory manifestations in CIA rats and reduced inflammatory and pyroptosis-associated responses in LPS/ATP-stimulated RAW264.7 cells. SXD treatment was accompanied by increased Pol β expression, attenuation of DNA-damage-associated changes, and suppression of cGAS/STING-, NF-κB-, and NLRP3-associated signaling. The reduced efficacy of SXD after Pol β knockdown supports partial involvement of Pol β-associated processes. Chebulagic acid, kaempferol, gentiopicroside, and berberine were identified as preliminary candidates and may represent major active constituents of SXD. Further studies should clarify their individual and combined contributions.

## Figures and Tables

**Table 1:** Chemical composition of serum from SXD-treated rats. Mass error was calculated as (measured m/z − theoretical m/z)/theoretical m/z × 10^6^.

No.	tR/min	Compounds	Formula	Theoretical m/z	Measured m/z	Ion Species	Mass Error (ppm)	Product Ions (m/z)
1	3.01	Betaine	C_5_H_11_NO_2_	118.0863	118.0868	[M+H]^+^	+4.23	59.0732
2	8.78	Gallic acid	C_7_H_6_O_5_	169.0142	169.0147	[M−H]^−^	+2.96	151.004
3	11.91	Loganic acid	C_16_H_24_O_10_	375.1297	375.1304	[M−H]^−^	+1.87	151.0780, 125.0612, 107.0435
4	13.65	Gentiopicroside	C_16_H_20_O_9_	355.1035	355.1049	[M−H]^−^	+3.94	193.0524, 149.0636
5	13.67	Ferulic acid	C_10_H_10_O_4_	193.0506	193.0512	[M−H]^−^	+3.11	134.0377, 149.0615, 178.0276
6	22.17	Magnoflorine	C_20_H_24_NO_4_	342.1705	342.1716	[M]^+^	+3.21	297.1137, 282.0904, 265.0872, 237.0926, 222.0690
7	58.95	Jatrorrhizine	C_20_H_20_NO_4_	338.1387	338.1373	[M]^+^	−4.14	280.0887, 294.1047, 308.0834, 322.0997
8	74.66	Berberine	C_20_H_18_NO_4_	336.1230	336.1221	[M]^+^	−2.68	320.0937, 306.0772, 292.0985
9	83.56	Quercetin	C_15_H_10_O_7_	303.0499	303.0501	[M+H]^+^	0.66	165.0190, 229.0509
10	84.17	Naringenin	C_15_H_12_O_5_	273.0757	273.0765	[M+H]^+^	2.93	153.0191
11	87.03	Kaempferol	C_15_H_10_O_6_	287.0550	287.0543	[M+H]^+^	−2.44	153.0183

## Data Availability

The data supporting the findings of this study are included in the article and its Supplementary Materials. The Supplementary Materials are deposited in Zenodo under https://doi.org/10.5281/zenodo.20829138 (accessed on 6 December 2025). Further inquiries may be directed to the corresponding authors.

## Supplementary Materials

The following supporting information can be downloaded at https://www.mdpi.com/article/10.3390/ph19081302/s1, Figure S1: Structural formulas of the prototype components of SXD absorbed into the bloodstream in rat serum. Figure S2: Inhibitory effects of eight active constituents on inflammatory cytokine release. Figure S3: HPLC chromatograms of mixed standards and samples. Figure S4: HPLC fingerprint of 20 batches of SXD. (R: reference fingerprint). Figure S5: Effects of different Pol β siRNA sequences on Pol β expression in macrophages. Figure S6: Therapeutic Effects of SXD in CIA Rats. Table S1: The detailed chromatographic conditions of HPLC analysis. Table S2: Sequences of primers for qRT-PCR. Table S3: Pol β RNA oligo sequence. Table S4: Method validation parameters for 4 active constituents. Table S5: Similarity data of 20 batches of SXD. Table S6. Quantification of 4 key active constituents in SXD (%).

## Abbreviations

The following abbreviations are used in this manuscript:

AI	Arthritis Index
ANOVA	One-way analysis of variance
BCA	Bicinchoninic Acid Assay
BER	Base Excision Repair
cGAS	Cyclic GMP–AMP synthase
DMARDs	Disease-Modifying Anti-Rheumatic Drugs
DMEM	Dulbecco’s Modified Eagle’s Medium
dsDNA	Double-Stranded DNA
ELISA	Enzyme-Linked Immunosorbent Assay
GO	Gene Ontology
GSDMD-N	N-terminal Fragment of Gasdermin D
KEGG	Kyoto Encyclopedia of Genes and Genomes
LDH	Lactate Dehydrogenase
LPS	Lipopolysaccharide
MMP	Matrix Metalloproteinase
MTX	Methotrexate
NF-κB	Nuclear Factor Kappa-B
NLRP3	NOD-Like Receptor Family Pyrin Domain Containing 3
NSAIDs	Non-Steroidal Anti-Inflammatory Drugs
PI	Propidium Iodide
RAW264.7	Murine macrophage-like cell line
SCFA	Short-Chain Fatty Acid
STING	Stimulator of Interferon Genes
TBK1	TANK-binding kinase 1
SEM	Scanning Electron Microscopy
TNF-α	Tumor Necrosis Factor Alpha
γH2AX	Phosphorylated Histone H2AX
