# Supplementary material for: Siweixizangmaoru Decoction Alleviates Rheumatoid Arthritis by Enhancing Pol β-Mediated Attenuation of DNA Damage and Suppression of cGAS/STING/NF-κB/NLRP3-Associated Pyroptosis
Source: Pharmaceuticals (Basel). 2026 Aug 18;19(8):1302. doi: 10.3390/ph19081302 (PMC13516318; doi:10.3390/ph19081302)
Supplement: Supplementary file 1 [file pharmaceuticals-19-01302-s001.zip › pharmaceuticals-4427110-supplementary.pdf]

Supporting Information for

Article

Siweixizangmaoru Decoction Alleviates Rheumatoid Arthritis by Enhancing Pol β-Mediated Attenuation of DNA Damage and Suppression of cGAS/STING/NF-κB/NLRP3-Associated Pyroptosis

Xiaotong Chu [1], Xiao Chen [1], Yanxiang Yuan [1], Yanfei Niu [1], Wentao Zhou [2], Yiming Cui [2], Yongyue Pan [2], Haifeng Liu [3], Zhuoma Dongzhi [2], Shan Huang [1,*] and Bin Li [1,2,*]

[1] Department of Pharmaceutical Engineering and Pharmaceutical Chemistry, School of Chemical Engineering, Qingdao University of Science and Technology, Qingdao 266042, China; 17205228560@163.com (X.C.)
[2] Department of Pharmacy, College of Medicine, Xizang University, Lhasa 850000, China; zmdz1980@utibet.edu.cn (Z.D.)
[3] College of Nursing, Qingdao Binhai University, Qingdao 266555, China
* Correspondence: huangshan@qust.edu.cn (S.H.); leebin009@163.com (B.L.)

Contents **Page**

1. Supplementary tables S3

Table S1. The detailed chromatographic conditions of HPLC analysis. S3

Table S2. Sequences of primers for qRT-PCR. S4

Table S3. Pol β RNA oligo sequence. S5

Table S4. Method validation parameters for 4 active constituents. S6

Table S5. Similarity data of 20 batches of SXD. S7

Table S6. Quantification of 4 key active constituents in SXD (%). S8

2. Supplementary figures S8

Figure S1. Structural formulas of the prototype components of SXD absorbed into the bloodstream in rat serum. S9

Figure S2. Inhibitory effects of eight active constituents on inflammatory cytokine release. S10-S11

Figure S3. HPLC chromatograms of mixed standards and samples. S11

Figure S4. HPLC fingerprint of 20 batches of SXD. (R: reference fingerprint). S12

Figure S5. Effects of different Pol β siRNA sequences on Pol β expression in macrophages. S12

Figure S6. Therapeutic Effects of SXD in CIA Rats. S13-S14

1. Supplementary tables

Table S1.

Time (min)	1% Phosphoric acid water (A)	Acetonitrile (B)	Detection wavelength:	Flow rate	Injection volume	Column temperature
0	97%	3%	245 nm	1 mL/min	5 μL	35℃
10	93%	7%				
25	90%	10%				
35	83%	17%				
60	75%	25%				
75	71%	29%				
95	55%	45%				
110	40%	60%				

The detailed chromatographic conditions of HPLC analysis.

Table S2.

Sequences of primers for qRT-PCR.

Gene	Forward primer sequences (5′—3′)	Reverse primer sequences (5′—3′)
Pol β	GAAACTGCCAGGAGTAGGAACAA	GCAGATGGTCCAATGCCAGTA
IL-18	GACTCTTGCGTCAACTTCAAGGA	TTTTGTCAACGAAGAGAACTTGGT
IL-1β	CTTTCCCGTGGACCTTCCA	CTCGGAGCCTGTAGTGCAGTT
β-actin	GGCTGTATTCCCCTCCATCG	CCAGTTGGTAACAATGCCATGT

Table S3.

Pol β RNA oligo sequence.

Target	Sense strand	Antisense strand
Pol β siRNA676	AGCAAACAGCCAAAGUUGUTT	ACAACUUUGGCUGUUUGCUTT
Pol β siRNA883	GGGAGUGACAUCUUUAAUATT	UAUUAAAGAUGUCACUCCCTT
Pol β siRNA470	AACGAAUUGGGCUGAAAUATT	UAUUUCAGCCCAAUUCGUUTT
Pol β siRNA64	AUGAGCAAACGCAAGGCGCTT	GCGCCUUGCGUUUGCUCAUTT

Table S4.

Method validation parameters for 4 active constituents.

Analytes	Precision RSD/ % (n = 6)	Repeatability RSD/ % (n = 6)	Accuracy (n = 6)	
			Mean	RSD/%
Gentiopicroside	0.77	1.98	99.74	0.20
Chebulagic acid	0.53	0.57	99.03	0.68
Berberine	0.17	0.94	99.24	0.53
Kaempferol	0.23	1.29	99.53	0.53

Table S5.

Similarity data of 20 batches of SXD.

ID	R	ID	R
S1	0.982	S11	0.976
S2	0.976	S12	0.981
S3	0.987	S13	0.985
S4	0.975	S14	0.978
S5	0.984	S15	0.977
S6	0.978	S16	0.989
S7	0.968	S17	0.986
S8	0.973	S18	0.993
S9	0.981	S19	0.988
S10	0.984	S20	0.974

Table S6.

Quantification of 4 key active constituents in SXD (%).

ID	Analytes (%)			
	Gentiopicroside	Chebulagic acid	Berberine	Kaempferol
S1	1.216	0.403	0.489	0.324
S2	1.234	0.467	0.458	0.375
S3	1.324	0.398	0.427	0.334
S4	1.359	0.453	0.557	0.388
S5	1.223	0.478	0.538	0.375
S6	1.117	0.399	0.455	0.322
S7	1.329	0.473	0.437	0.319
S8	1.234	0.434	0.476	0.367
S9	1.267	0.454	0.579	0.313
S10	1.095	0.445	0.446	0.298
S11	1.189	0.432	0.438	0.378
S12	1.321	0.458	0.599	0.345
S13	1.117	0.421	0.534	0.321
S14	1.354	0.429	0.562	0.333
S15	1.123	0.394	0.519	0.354
S16	1.228	0.433	0.456	0.308
S17	1.119	0.456	0.437	0.302
S18	1.239	0.472	0.555	0.343
S19	1.116	0.489	0.428	0.322
S20	1.127	0.421	0.556	0.345
Average (%)	1.217	0.44	0.497	0.338

2. Supplementary figures

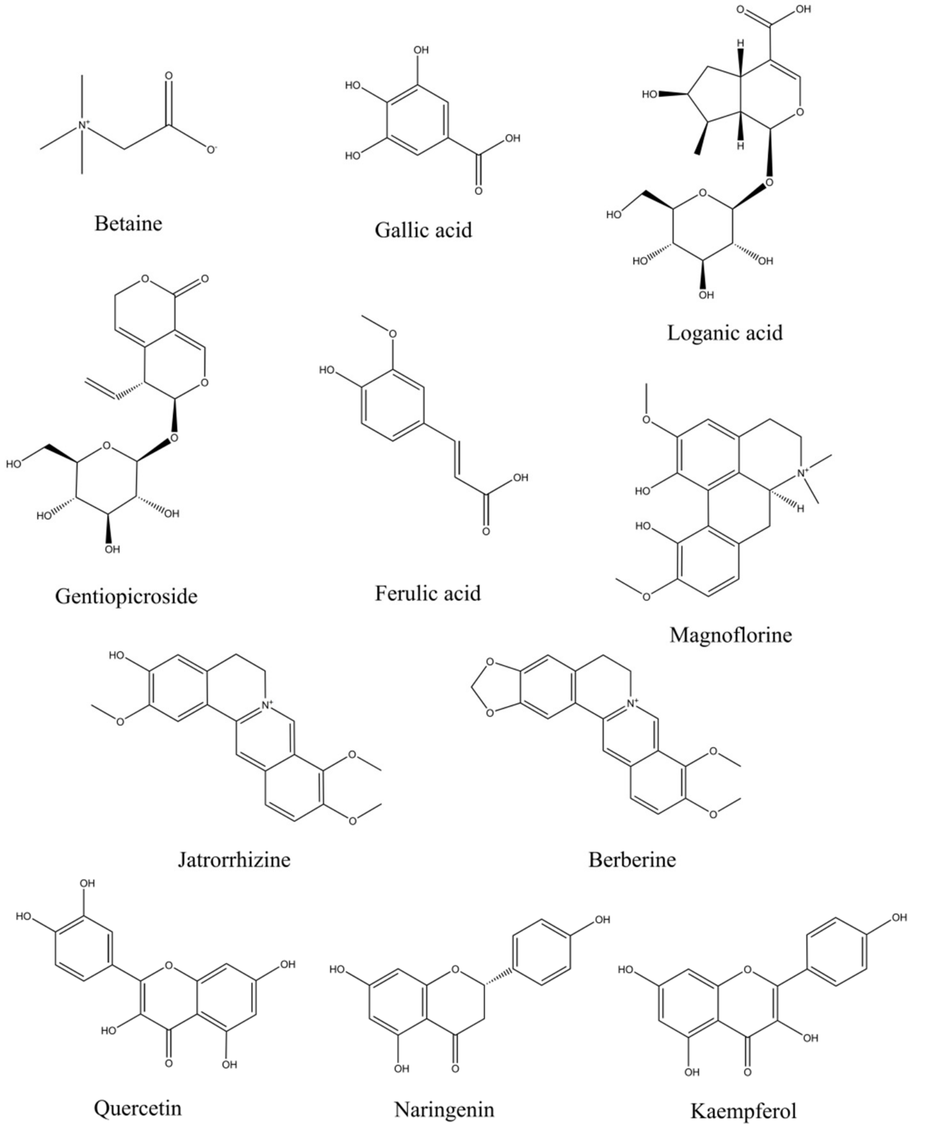

Figure S1. Structural formulas of the prototype components of SXD absorbed into the bloodstream in rat serum.

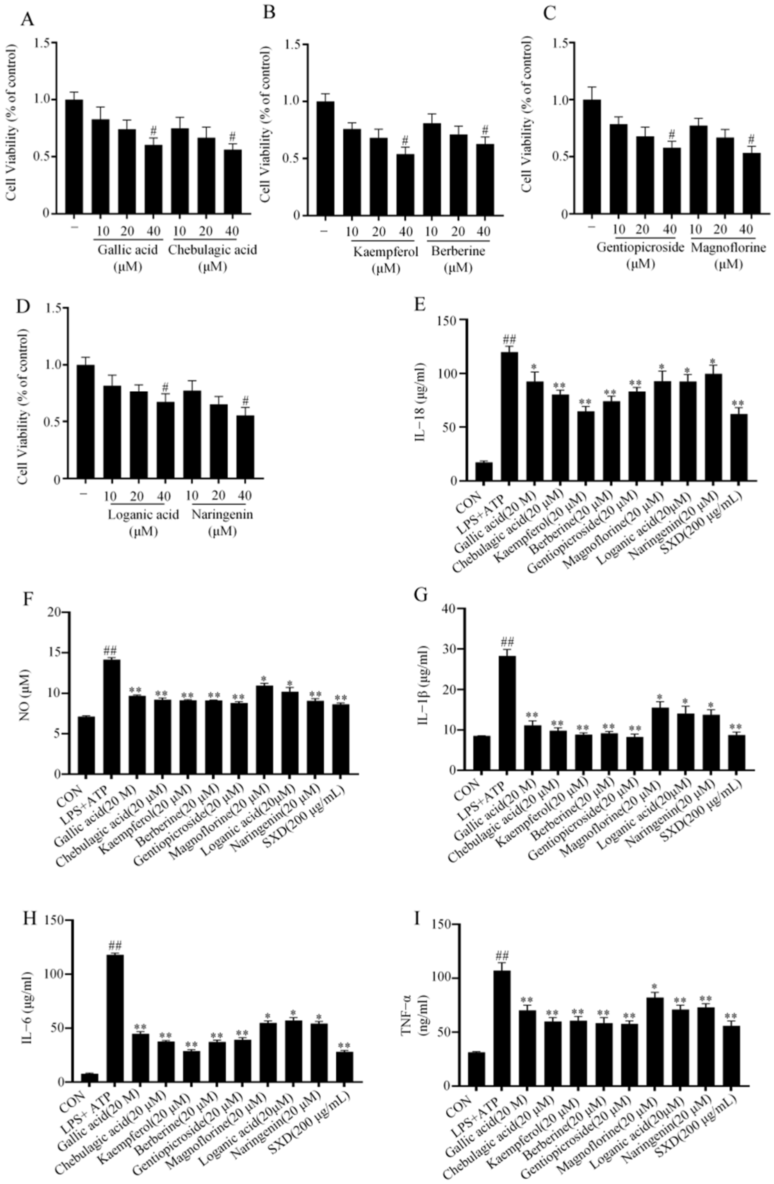

Figure S2. Inhibitory effects of eight active constituents on inflammatory cytokine release. (**A-D**) Screening of the optimal concentrations of the eight active constituents. (**E-I**) Effects of the eight active constituents on the release of inflammatory mediators, including IL-18, NO, IL-1β, IL-6, and TNF-α. Data represent mean ± SD of three biological independent experiments. *p<0.05, **p<0.01 vs. the LPS/ATP group. ##p<0.01 vs. the CON group.

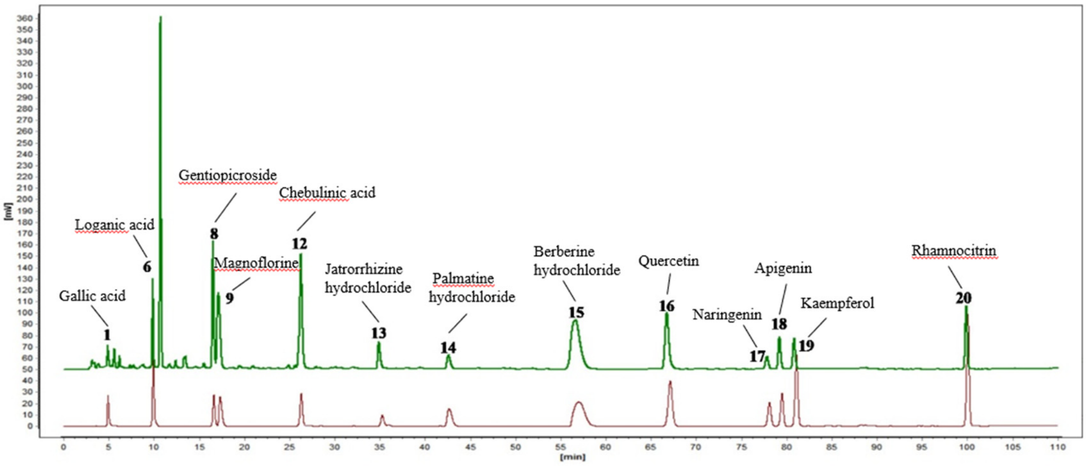

Figure S3. HPLC chromatograms of mixed standards and samples.

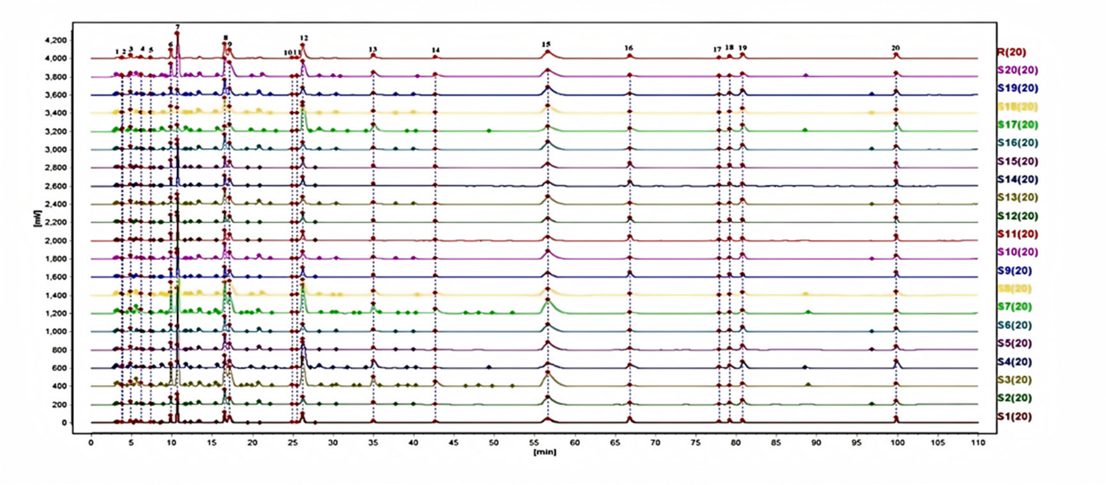

Figure S4. HPLC fingerprint of 20 batches of SXD. (R: reference fingerprint).

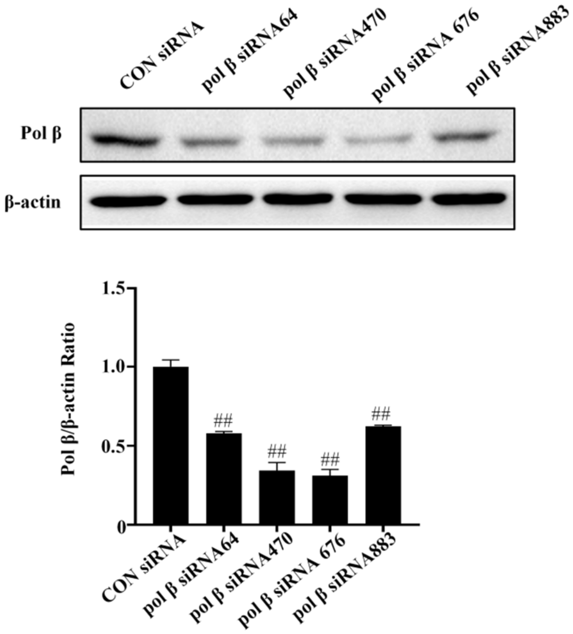

Figure S5. Effects of different Pol β siRNA sequences on Pol β expression in macrophages.

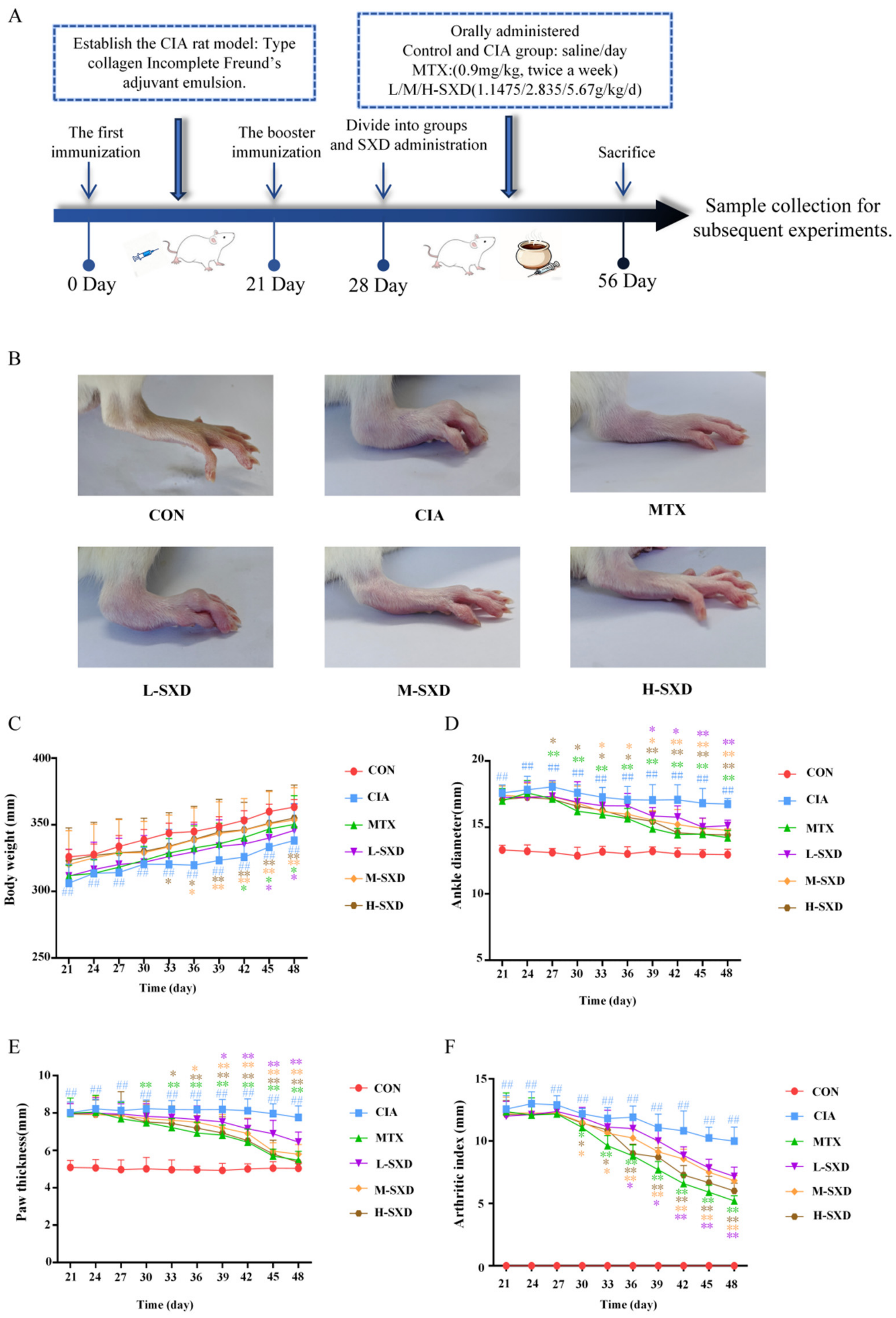

Figure S6. Therapeutic Effects of SXD in CIA Rats. (**A**) Experimental design. (**B**) Representative pictures of hind paws were showed. (**C**) The body weight changes of rats. (**D**) The ankle joint thickness of rats. (**E**) The paw thickness of rats. (**F**) The arthritis score of rats. Data are expressed as mean ± SD of six independent experiments. Comparisons between groups at the same time points showed significant differences. Specifically, under identical Time (day) conditions: *$p<0.05$, **$p<0.01$ vs. the CIA group. #$p<0.05$, ##$p<0.01$ vs. the CON group.
